# Statistical Optimization of Graphene Nanoplatelet-Reinforced Epoxy Nanocomposites via Box–Behnken Design for Superior Flexural and Dynamic Mechanical Performance

**DOI:** 10.3390/polym17233218

**Published:** 2025-12-03

**Authors:** Júlia Mendes, Camila Prudente Magalhães, Letícia Vitorazi, Noemi Raquel Checca Huaman, Sergio Neves Monteiro, Teresa Gómez-del Río, Ulisses Oliveira Costa

**Affiliations:** 1 Materials and Metallurgical Engineering Department, Federal Fluminense University, Rio de Janeiro 27255-125, Brazil; juliamendes@id.uff.br (J.M.); camila_prudente@id.uff.br (C.P.M.); leticiavitorazi@id.uff.br (L.V.); 2 Brazilian Center for Physics Research, CBPF, Rio de Janeiro 22290-180, Brazil; noemiraquelchecca@gmail.com; 3 Materials Science Department, Military Institute of Engineering (IME), Rio de Janeiro 22290-270, Brazil; snevesmonteiro@gmail.com; 4 Grupo de Durabilidad e Integridad Mecánica de Materiales Estructurales (DIMME), Universidad Rey Juan Carlos, 28933 Madrid, Spain; mariateresa.gomez@urjc.es

**Keywords:** graphene nanoplatelets, epoxy nanocomposites, Box–Behnken design, flexural properties

## Abstract

Graphene nanoplatelets (GNPs) are efficient nanofillers for improving the mechanical and thermal properties of epoxy resins due to their high stiffness, aspect ratio, and interfacial reinforcement ability. This study employs a three-factor, three-level Box–Behnken Design (BBD) to investigate the combined effect of GNP content (0.5–3.5 wt.%), hardener concentration (9–17 phr), and post-curing temperature (30–120 °C) on DGEBA/TETA epoxy nanocomposites. Mechanical, thermal, dynamic mechanical, and morphological characterizations (flexural testing, DMA, TGA, DSC, FTIR, SEM, TEM, and AFM) established structure–property correlations. The optimized formulation (2.0 wt.% GNP, 9 phr hardener, and 120 °C post-curing) exhibited superior reinforcement, with flexural strength of 322.0 ± 12.8 MPa, flexural modulus of 9.7 ± 0.5 GPa, and strain at break of 4.4 ± 0.2%, corresponding to increases of 197%, 155%, and 91% compared with neat epoxy. DMA confirmed a rise in storage modulus from 2.9 to 7.5 GPa and a Tg of 143 °C, while TGA showed a 15 °C improvement in thermal stability. Statistical analysis identified post-curing temperature as the dominant factor governing Tg, stiffness, and thermal stability, with synergistic contributions from GNP content and hardener concentration to the overall network performance. These results surpass those of GO- and CNT-based systems, demonstrating the superior efficiency of GNPs under optimized conditions. The proposed approach provides a robust pathway for developing epoxy nanocomposites with low filler content and enhanced multifunctional performance.

## 1. Introduction

Graphene-based nanocomposites have emerged as transformative materials for next-generation structural systems, offering an unprecedented balance of mechanical robustness, thermal stability, and multifunctionality. This potential arises from the unique two-dimensional architecture and extraordinary intrinsic properties of graphene and its derivatives, including high Young’s modulus, exceptional electrical and thermal conductivity, and impermeability to gases [1,2,3]. These attributes have inspired the development of graphene-based polymer nanocomposites tailored for applications ranging from aerospace and energy storage to protective coatings and innovative structures. Within this class, graphene nanoplatelets (GNPs) stand out as cost-effective and processable nanofillers that can substantially enhance the stiffness, toughness, and thermal stability of thermoset matrices, such as epoxy resins, even at very low concentrations [4,5,6,7].

Recent investigations have confirmed that GNP incorporation, typically in the range of 0.1–0.5 wt.%, produces synergistic improvements in tensile, compressive, and thermal responses due to crack deflection, constrained polymer chain mobility, and efficient load transfer across the filler–matrix interface [8,9,10,11]. These effects position GNP-reinforced epoxies among the most promising candidates for lightweight, high-performance systems. However, realizing their full potential remains a complex challenge. Beyond specific critical loadings (generally above 1–1.5 wt.%), dispersion quality deteriorates, leading to nanoparticle agglomeration, microvoid formation, and interfacial stress concentration that compromise mechanical integrity [12,13]. Achieving homogeneous dispersion while maintaining optimal crosslinking density thus remains a key barrier to scalability.

Another major limitation in the literature lies in the narrow experimental scope of most studies. Investigations frequently adopt a univariate approach, varying a single processing or formulation factor, such as GNP content, curing temperature, or hardener ratio, while keeping the others constant [5,14,15,16]. This reductionist strategy neglects the nonlinear and often synergistic interactions among these variables, which critically influence the thermomechanical performance of epoxy nanocomposites. For example, Wei et al. [12] demonstrated that aggregation becomes significant beyond 1 wt.% GNP, while Agustina et al. [13] emphasized the interplay between platelet size, dispersion state, and mechanical reinforcement efficiency. Despite the growing use of improved exfoliation and mixing strategies [15], few studies systematically explore how processing parameters jointly modulate structure–property relationships, particularly at higher GNP concentrations, where competition between improved load transfer and increased defect density governs the final performance.

In this regard, the use of statistical design and optimization techniques has gained traction as a powerful means to model and predict the multivariable interactions intrinsic to polymer nanocomposites. Response Surface Methodology (RSM) and related Design-of-Experiments (DoE) tools enable systematic exploration of multiple factors simultaneously, allowing for the identification of optimal processing windows and predictive response models [14,17,18,19]. Moreover, advanced frameworks integrating RSM with machine learning (ML) and artificial intelligence (AI) have recently been proposed to enhance parameter optimization and minimize experimental cost [18]. Such approaches have been successfully implemented in metallic and polymeric systems but remain rare in graphene-reinforced epoxy nanocomposites, especially under high filler loadings or variable curing conditions. Notably, Hussain et al. [20] applied a DoE-based framework to toughen epoxy matrices by combining block copolymers and GNPs, revealing promising trends but still constrained to relatively low graphene concentrations. Likewise, Pereira et al. [16] highlighted the potential of GNPs for developing multifunctional epoxy systems, yet did not address post-curing or stoichiometric effects, parameters essential for achieving stable crosslinked networks.

Additionally, hybrid filler strategies and interfacial modifications continue to attract attention as means to overcome the limitations of single-filler systems. Studies combining GNPs with nanoclays, nanotubes, or oxides have demonstrated synergistic effects that improve both mechanical and thermal responses, while surface functionalization strategies, such as silanization or amine grafting, promote interfacial compatibility and stress transfer [6,7]. Nevertheless, these enhancements are strongly influenced by curing kinetics and hardener composition, underscoring the need for a comprehensive understanding of processing–structure–property correlations under multivariate conditions.

Against this backdrop, the present study introduces a statistically grounded and systematically validated framework for the multivariable optimization of epoxy nanocomposites reinforced with graphene nanoplatelets (GNPs). A three-factor, three-level Box–Behnken Design (BBD) was employed to elucidate the individual and interactive effects of GNP content (0.5–3.5 wt.%), hardener concentration (9–17 phr), and post-curing temperature (30–120 °C) on the functional performance of DGEBA/TETA epoxy nanocomposites. The materials were comprehensively characterized through thermal (DSC, TGA), mechanical–dynamic (flexural and DMA), morphological (SEM, TEM, AFM), and chemical (FTIR) analyses, establishing quantitative structure–property correlations. This integrative experimental–statistical approach bridges a critical gap in the literature by providing a predictive, rational pathway for designing GNP/epoxy nanocomposites with enhanced mechanical and thermal performance, tailored for structurally demanding, multifunctional engineering applications.

## 2. Materials and Methods

### 2.1. Materials

The epoxy-based nanocomposites were produced from a two-component system comprising bisphenol A diglycidyl ether (DGEBA, grade MC130) and triethylenetetramine (TETA, grade FD129), both supplied by Epoxyfiber^®^ (Rio de Janeiro, Brazil). The resin and curing agent were mixed at three mass ratios: 100:9, 100:13, and 100:17 (resin:hardener). GNP powder (grade UGZ1004) was obtained from UCSGraphene (Rio Grande do Sul, Brazil) and incorporated into the epoxy matrix.

For processing and characterization, a high-speed mechanical stirrer (Fisatom, model 713D, São Paulo, Brazil) operating at 1500 rpm was used to homogenize the formulations. The curing and post-curing steps were carried out in a temperature-controlled oven, and the mechanical properties were assessed using a universal testing machine.

### 2.2. Methods

#### 2.2.1. Nanocomposite Preparation

The DGEBA epoxy resin was initially mixed with GNP at concentrations of 0.5, 2.0, and 3.5 wt.%. Homogenization was performed using a mechanical stirrer at room temperature (RT) for 15 min to ensure uniform dispersion. Subsequently, the TETA hardener was added under gentle stirring at RT, followed by vacuum degassing for 10 min to minimize bubble formation. The mixtures were then cast into silicone molds and cured at room temperature under controlled conditions. A post-curing process was subsequently conducted after approximately 24 h to complete the crosslinking reaction and enhance the final material properties.

#### 2.2.2. Experimental Design

A Box–Behnken Design (BBD) with three factors at three levels was adopted to investigate how the main processing parameters affect the mechanical, thermal, and morphological responses of the graphene-reinforced epoxy nanocomposites. The independent variables considered were the GNP loading (wt.%), the hardener content (wt.%), and the post-curing temperature (°C), as summarized in Table 1.

The factor levels shown in Table 1 were defined by combining preliminary screening experiments with information from our previous BBD-based optimization study on GNP/epoxy nanocomposites [12] and with general processing guidelines for DGEBA/TETA systems. GNP contents below 0.5 wt.% produced only modest improvements in stiffness and strength, whereas contents above approximately 3.5–4.0 wt.% led to excessive viscosity, visible agglomeration during mixing, and difficulties in obtaining defect-free specimens; therefore, 0.5–3.5 wt.% was chosen as a practical range that still allows significant reinforcement without compromising processability. The central hardener level corresponds to the supplier’s recommended nominal stoichiometric ratio. At the same time, the lower and upper bounds (9 and 17 phr) represent moderately under- and over-stoichiometric conditions that can modify network structure and Tg without reaching extreme plasticization or gelation. For the post-curing temperature, 30 °C represents curing close to room temperature, whereas 120 °C was selected as an upper limit above the initial Tg range and within the safe thermal window indicated by DSC/TGA screening and manufacturer recommendations, with intermediate levels chosen to capture the transition from under-cured to highly crosslinked networks.

The BBD strategy was selected because it allows evaluating curvature and interaction effects among variables with a relatively small number of experiments. The design matrix included replicated runs at the center point to improve the estimation of experimental error and increase the reliability of the fitted response surfaces. This experimental design provided a consistent basis for quantifying the combined influence of the processing parameters while efficiently using materials and laboratory time.

The variables were coded using the following equation:

c_i_ = (t_i_ − t_m_)/δ (1)

where t_i_ represents the weight fraction of factor i (i = 1 for GNPs; i = 2 for hardener content) and t_3_ is for post-curing temperature; c_i_ is the coded value of t_i_; t_m_ is the average parameter value; and δ is the interval |t_i_ − t_m_|. The levels of each factor presented in Table 1 were based on the mass of epoxy resin for i = 1 and i = 2.

Coded variables:

Response = (c_1_,c_2_,c_3_) = a + b_1_c_1_ + b_2_c_2_ + b_3_c_3_ + b_4_c_1_c_2_ + b_5_c_1_c_3_ + b_6_c_2_c_3_ + b_7_c_1_^2^ + b_8_c_2_^2^ + b_9_c_3_^2^ (2)

Original variables:

Response = (t_1_,t_2_,t_3_) = a + β_1_t_1_ + β_2_t_2_ + β_3_t_3_ + β_4_t_1_t_2_ + β_5_t_1_t_3_ + β_6_t_2_t_3_ + β_7_t_1_^2^ + β_8_t_2_^2^ + β_9_t_3_^2^ (3)

In these equations, $c_{1},c_{2},c_{3}$ and $t_{1},t_{2},t_{3}$ represent the coded and actual (uncoded) values of the independent variables, respectively. The term a corresponds to the regression intercept, while $b_{i}$ and $\beta_{i}$ are the regression coefficients associated with the effects of factor i. These models were used to construct the response surfaces and to analyze how each processing parameter influences the behavior of the nanocomposite films.

The experimental matrix shown in Table 2 was defined to adequately capture both the main (individual) and interaction effects of the selected factors, thereby enabling the identification of processing conditions that maximize the performance of the nanocomposites.

Statistical analysis of the BBD models was carried out using OriginLab^®^ software (version 2025b). The goodness-of-fit between the experimental responses and the regression surfaces was assessed using the coefficient of determination (R^2^). To penalize models with an excessive number of terms relative to the available experimental runs, the adjusted R^2^ was also computed. In contrast, the predicted R^2^ was used to verify the extrapolative capability of the models for conditions not included in the fitting dataset.

The overall significance of each model, as well as the contribution of the linear, quadratic, and interaction terms, was assessed by Analysis of Variance (ANOVA). Terms with *p*-values lower than 0.05 were regarded as statistically significant. The F-statistic provided the ratio of the variance explained by the model to the residual variance. At the same time, the Lack-of-Fit test was used to detect potential inadequacies in the regression model. In addition, the residuals’ standard deviation was calculated to quantify the scatter of the experimental data around the fitted response surfaces.

Based on these criteria, the fitted models were validated and subsequently used to reliably interpret the individual and combined effects of G, H, and T on the thermal and dynamic mechanical behavior of the epoxy nanocomposites.

#### 2.2.3. Nanocomposite Characterization

##### Mechanical Behavior

Flexural tests were performed according to the ASTM D790-17 standard [21] using a universal testing machine (EMIC DL2000) equipped with a 25 kN load cell. The test was conducted in a three-point bending configuration with a support span of 51.2 mm, corresponding to a span-to-depth ratio of 16:1 for specimens measuring 127 × 12.7 × 3.2 mm. The crosshead speed was set to 1.37 mm/min, which corresponds to a strain rate of 0.01 mm/mm/min (Procedure A). Tests were performed under ambient conditions. The mechanical properties assessed include: flexural modulus (MOE), determined from the slope of the initial linear portion of the load–deflection curve; flexural strength (MOR), defined as the maximum stress sustained by the specimen during the bending test; and flexural strain at break (ε_f_), calculated from the maximum deflection before failure, indicating the material’s ductility under bending.

##### Thermal Behavior

The thermal stability and decomposition behavior of the samples were evaluated in accordance with ASTM E1131-20 [22] using a NETZSCH STA 409 PC thermogravimetric analyzer (NETZSCH, Selb, Germany). Measurements were carried out under a nitrogen atmosphere, from 20 to 700 °C, applying a constant heating rate of 10 °C/min. The onset decomposition temperature (Tonset) was obtained from the derivative thermogravimetric (DTG) curves.

The glass transition temperature (Tg) was determined by differential scanning calorimetry (DSC) using a STAR SYSTEM DSC 1. The samples were heated from 20 to 300 °C at 10 °C/min under nitrogen flow, and Tg was determined from the corresponding DSC curves.

##### Dynamic Behavior

The dynamic mechanical behavior of the GNP/epoxy nanocomposites was evaluated using Dynamic Mechanical Analysis (DMA) in accordance with ASTM D7028-08 [23]. The tests were conducted on a Q800 DMA instrument (TA Instruments) at the Durability and Mechanical Integrity of Structural Materials Group (DIMME), Rey Juan Carlos University, Spain. The analyses were performed under a nitrogen atmosphere at a heating rate of 5 °C/min, a frequency of 1 Hz, and a temperature range of 20 to 180 °C. The storage modulus (E’), loss modulus (E”), and damping factor (tanδ) were determined from the resulting data. The specimens used in the three-point bending mode had dimensions of 35 × 13 × 1.75 mm.

##### Morphological and Structural Analysis

The morphology of the materials was investigated by Scanning Electron Microscopy (SEM), Transmission Electron Microscopy (TEM), and Atomic Force Microscopy (AFM). SEM observations were carried out on a Quanta FEG 250 field-emission microscope (FEI, São Bernardo do Campo, Brazil) to examine the fracture surfaces of the nanocomposites after tensile testing. These images were also used to compare the morphology of the GNPs with that of the original graphite powder, emphasizing differences in surface texture and exfoliation. Before SEM imaging, the samples were coated with a thin gold layer under high vacuum using a LEICA EM ACE600 sputter coater (Leica, Wetzlar, Germany) to enhance surface conductivity and image quality.

To gain further insight into the structure of the GNPs, TEM analyses were performed on a JEOL 2100F high-resolution transmission electron microscope operated at 200 kV. A diluted GNP dispersion was drop-cast onto an ultrathin carbon film supported on a Lacey grid. Bright-field (BF) imaging and selected-area electron diffraction (SAED) were used to characterize the nanosheet morphology and crystalline structure. Electron Energy Loss Spectroscopy (EELS), focused on the carbon K-edge, was also used to probe the electronic structure and defect-related features of the GNPs.

AFM measurements were conducted with an Easyscan 2 FlexAFM system (Nanosurf, Langen, Germany) operating in tapping mode with a TAP 190AL-G cantilever. This technique provided high-resolution topographic maps and thickness measurements of individual GNP flakes, enabling nanoscale assessment of exfoliation efficiency and surface features.

##### Chemical Properties

Fourier-Transform Infrared Spectroscopy (FTIR) was employed to evaluate the chemical interactions and potential structural modifications in the epoxy nanocomposites as a function of processing conditions. Spectra were acquired in the range of 4000–500 cm^−1^ using a Bruker Alpha II spectrometer (Billerica, MA, USA) equipped with an attenuated total reflectance (ATR) accessory. Each sample from the BBD matrix was analyzed in triplicate to ensure reproducibility, and the resulting spectra were subjected to both univariate and multivariate statistical analyses.

To assess the individual effects of GNP content, hardener concentration, and post-curing temperature on the nanocomposites’ chemical features, characteristic absorption bands were identified and quantified. Specific attention was paid to functional groups relevant to the epoxy network, including the oxirane ring (~915 cm^−1^), ether groups (~1240 cm^−1^), aromatic skeleton vibrations (~1500–1600 cm^−1^), and hydroxyl groups (~3400 cm^−1^). For each selected band, both peak intensity and integrated area were measured using the OriginLab^®^ software (version 2025b) with baseline correction.

## 3. Results and Discussion

### 3.1. Thermal Behavior

Thermal analysis of the GNP/epoxy nanocomposites was carried out by combining DSC and TGA. From these measurements, the glass transition temperature (Tg), the onset of thermal degradation (Tonset), the temperature of maximum degradation rate (Tmax), and the residual exothermic enthalpy associated with post-curing (ΔH_res_) were obtained for all formulations. The corresponding values are summarized in Table 3. Since all these thermal parameters are sensitive to the processing conditions (G, H, and T), they were further analyzed to evaluate the combined effects of these factors on the network development and thermal stability of the nanocomposites.

**Figure 1:**
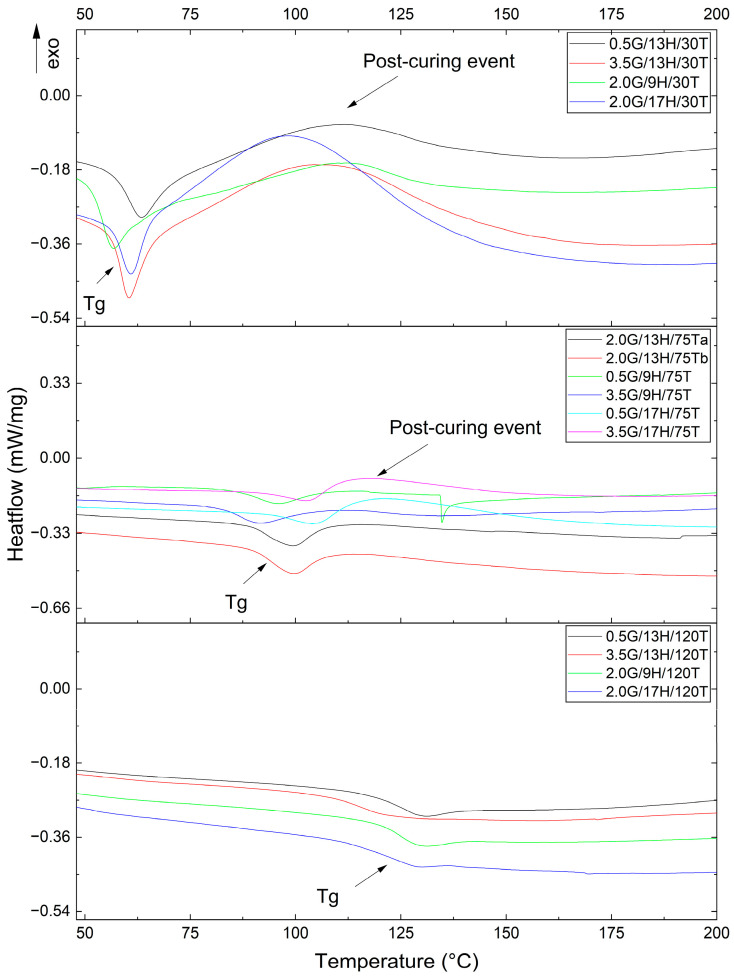
DSC curves for the nanocomposites grouped by post-curing temperature.

A clear dependence of Tg on post-curing temperature was observed, allowing the identification of three distinct regimes. Samples post-cured at 30 °C exhibited markedly low Tg values (≈50–55 °C), consistent with an incompletely crosslinked network that retains significant molecular mobility, leading to post-curing exothermic events, as shown in Figure 1 [24]. At an intermediate post-curing temperature of 75 °C, the Tg values increased substantially (≈80–95 °C) substantially, indicating a more advanced degree of crosslinking and partial restriction of chain motion [24]. Finally, samples post-cured at 120 °C reached the highest Tg values (>110 °C), confirming the formation of a highly crosslinked, rigid polymer network.

Figure 1 reinforces these trends, as the step-like transitions corresponding to Tg progressively shift to higher temperatures and become more defined with increasing post-curing temperature. This behavior is typical of epoxy systems, in which additional curing reactions occur during post-treatment, leading to greater network consolidation [25]. The well-separated Tg regimes emphasize the dominant influence of post-curing temperature over the other variables studied (GNP and H), establishing it as the key parameter for tailoring the viscoelastic response of the nanocomposites. From a mechanistic standpoint, the predominance of T over G and H can be rationalized by considering that Tg in amine-cured epoxies is primarily governed by the extent of network formation and the associated reduction in free volume. Higher post-curing temperatures allow the system to overcome diffusion limitations and to complete the epoxy–amine conversion, whereas variations in stoichiometry and GNP loading only modulate this process secondarily [26]. In diffusion-controlled regimes, GNPs may locally slow the reaction by adsorbing amine groups and acting as thermal barriers. Still, once a highly crosslinked network is established, its main contribution becomes the physical restriction of segmental motion through interfacial π–π and hydrogen-bonding interactions. Consequently, the most significant changes in Tg arise from thermally activated crosslinking, with GNPs acting mainly as kinetic and mechanical modifiers of a temperature-driven curing process [27].

The thermal transition behavior of the GNP/epoxy nanocomposites was further investigated using BBD to identify the most influential processing parameter on Tg. The regression analysis yielded an excellent fit, with R^2^ = 0.92, R^2^adj = 0.91, and R^2^pred = 0.88, confirming the model’s predictive adequacy. The lack-of-fit test was not significant (*p* = 0.1049), indicating that the full-quadratic model with backward elimination at alpha = 0.05 effectively fits the experimental data with minimal unexplained variation.

As shown by the ANOVA, the T exerted a highly significant effect on Tg (F = 147.11, *p* < 0.0001). At the same time, the contributions of GNP content and H were statistically negligible. The BBD confirms that temperature is the dominant factor, overshadowing all other variables. This linear dependence reflects the strong correlation between the degree of cure and the post-curing temperature, a typical behavior for epoxy systems where higher temperatures promote further crosslinking reactions and network densification [25].

The BBD shows an apparent monotonic increase in Tg with T, from approximately 85 °C at 30 °C curing to around 110–115 °C at 120 °C post-cure, confirming the enhancement in chain rigidity and reduction in molecular mobility. This effect arises from both chemical crosslinking and secondary interactions (e.g., π–π stacking and hydrogen bonding) between the epoxy network and GNP, which together restrict segmental relaxation [25].

Beyond the glass transition, the DSC thermograms exhibited additional exothermic peaks associated with post-curing reactions that occurred within the instrument during heating. The integration of these exothermic events provided residual enthalpy values (ΔH_res_, J g^−1^) that serve as indicators of the degree of conversion achieved before the analysis, larger ΔH_res_ values reflecting lower pre-cure extents. A pronounced dependence on post-curing temperature was observed: specimens treated at 30 °C exhibited the highest ΔH_res_ (≈6–9 J g^−1^), revealing significant unreacted epoxy fractions; those cured at 75 °C presented intermediate values (≈0.4–2.4 J g^−1^); while samples post-cured at 120 °C displayed nearly negligible exothermic events, confirming extensive network completion before DSC testing [25,28]. At 75 °C, increasing the hardener content from 9 to 17 phr led to a systematic rise in ΔH_res_ (e.g., 0.381 to 2.365 J g^−1^ for 0.5 wt.% GNP; 0.896 to 1.704 J g^−1^ for 3.5 wt.% GNP), suggesting that excess amine and partial vitrification at this temperature constrained diffusion and postponed reaction completion. The influence of GNP content was comparatively secondary. Still, it was condition-dependent: Under low post-curing temperatures (30 °C), higher GNP loading increased ΔH_res_, plausibly due to amine adsorption and the barrier effect of nanoplatelets on heat transfer and molecular mobility; however, at 75 °C, this trend was less pronounced and non-monotonic [25,26,27,28,29]. These findings corroborate the evolution of Tg and the secondary exothermic features observed in the thermograms (Figure 1), reinforcing that T is the dominant factor governing residual cure, followed by H, while GNP acts as a kinetic modulator that influences network completion under diffusion-limited regimes [25].

**Figure 2:**
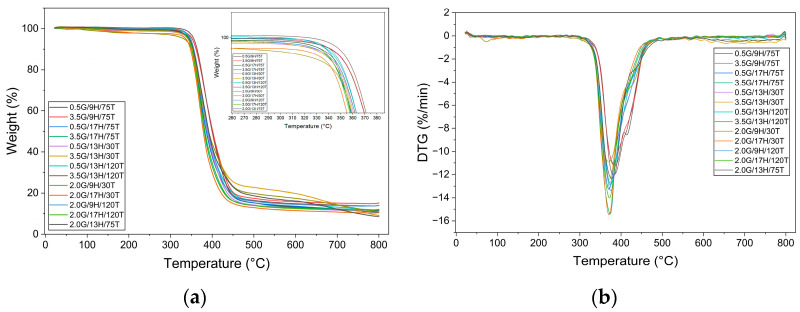
TGA curves for all the GNP-reinforced epoxy nanocomposites: (**a**) TG, and (**b**) DTG.

The thermal stability of the GNP/epoxy nanocomposites was further assessed through TGA, from which the Tonset and the Tmax were derived (Table 3) and (Figure 2). In contrast to Tg, which showed strong sensitivity to post-curing conditions, both Tonset and Tmax exhibited more modest variations across the investigated parameter space. However, meaningful trends could still be identified.

The Tonset values ranged from 342.2 °C to 352.4 °C, indicating that all formulations maintained a relatively high level of initial thermal stability. Overall, Tonset appeared less sensitive to the processing parameters compared to Tg. Nevertheless, some patterns emerged. Samples post-cured at 120 °C tended to exhibit slightly higher Tonset values (e.g., 352.4 °C for 3.5G/13H/120T and 347.4 °C for 2.0G/9H/120T), suggesting that higher crosslinking densities delay the onset of degradation [28]. By contrast, lower values were consistently observed in systems with high hardener content (H = 17 phr) or low post-curing temperature (30 °C), indicating that excessive amine or incomplete network development can facilitate earlier degradation [29]. The effect of GNP content alone was not systematic, implying that its influence is strongly modulated by the interplay with curing chemistry and thermal treatment.

The Tmax values fell within a narrow interval of 369.3–385.1 °C, confirming that this parameter is comparatively less sensitive to compositional changes. Nonetheless, tendencies were observed. The highest Tmax (385.1 °C) was achieved for the sample with low GNP (0.5 wt.%), low hardener content (9 phr), and post-curing at 75 °C, indicating that intermediate thermal treatment combined with a balanced stoichiometry can maximize thermal stability at the peak of decomposition [30]. Conversely, the lowest Tmax (369.3 °C) was associated with a formulation post-cured at 30 °C, reinforcing the detrimental effect of insufficient network consolidation. A general trend was identified whereby increasing hardener concentration reduced Tmax, regardless of GNP level or post-curing temperature. This suggests that excess hardener may create local heterogeneities or residual reactive groups, thereby compromising thermal resistance [31]. The effect of GNP content was again non-monotonic: depending on the curing conditions, higher nanoparticle loading either slightly enhanced or reduced Tmax, consistent with its dual role as both a potential thermal barrier and a source of interfacial catalytic sites.

Together, these results demonstrate that while Tonset and Tmax remain relatively stable across formulations, their variations highlight the importance of processing control. Post-curing temperature exerts a positive effect by enhancing crosslink density, whereas high hardener contents systematically lower thermal stability, both at the onset and at the maximum degradation rate. The influence of GNP content is more complex and context-dependent, reinforcing the need for multivariable statistical analysis to disentangle synergistic and antagonistic effects.

### 3.2. Mechanical Behavior

#### Flexural Properties

The flexural properties of epoxy nanocomposites reinforced with GNPs, summarized in Table 4, demonstrate a wide variation in mechanical behavior as a function of the processing parameters (G, H, and T). Specifically, Table 4 reports the MOR, MOE, and ε_f_ obtained from three-point bending tests. These parameters were determined from stress–strain curves using Origin software, ensuring consistent and accurate evaluation of the experimental conditions established by the BBD.

Overall, the MOR values ranged from 180.7 MPa to 322.0 MPa, indicating substantial differences in flexural strength as a function of GNP content, hardener content, and post-curing temperature. The highest MOR (322.0 ± 12.8 MPa) was observed for the sample containing 2.0 wt.% GNP, 9 phr of hardener, and post-cured at 120 °C, suggesting that optimal nanoparticle dispersion and matrix reinforcement can be achieved under specific processing conditions [32].

The MOE varied between 8.3 ± 0.4 GPa and 12.6 ± 1.0 GPa. Higher MOE values were generally associated with lower hardener content and intermediate post-curing temperatures (~75 °C), which may be attributed to reduced nanoparticle agglomeration and more effective stress transfer within the polymer network [32].

Regarding ε_f_, values ranged from 1.9 ± 0.1% to 4.4 ± 0.2%. Samples exhibiting higher ε_f_ values, particularly the formulation with 2.0 wt.% GNP, low H content, and high T, demonstrated enhanced deformation capacity before failure, which may be advantageous for applications requiring improved toughness [32].

The analysis of MOR initially demonstrated that the proposed second-order model explained 55.0% of the experimental variability (R^2^ = 0.55), with an adjusted R^2^ of 0.50 and a predicted R^2^ of 0.43. While these values suggested a moderately adequate model, the lack-of-fit test was statistically significant (*p* = 0.0052), indicating that one or more points deviated from the general trend expected for the Box–Behnken design. Inspection of the residuals revealed that the formulation 2.0 wt.% GNP/17 phr/30 °C (MOR = 180.7 ± 6.7 MPa) behaved as an anomalous point, exhibiting the lowest flexural strength among all samples with 2.0 wt.% GNP and the most significant studentized residual and Cook’s distance. This condition corresponds to a highly unfavorable combination of factors in the design space, coupling a relatively high GNP content with the highest hardener content and the lowest curing temperature, which is expected to maximize viscosity, hinder filler dispersion, and severely limit network development.

**Figure 3:**
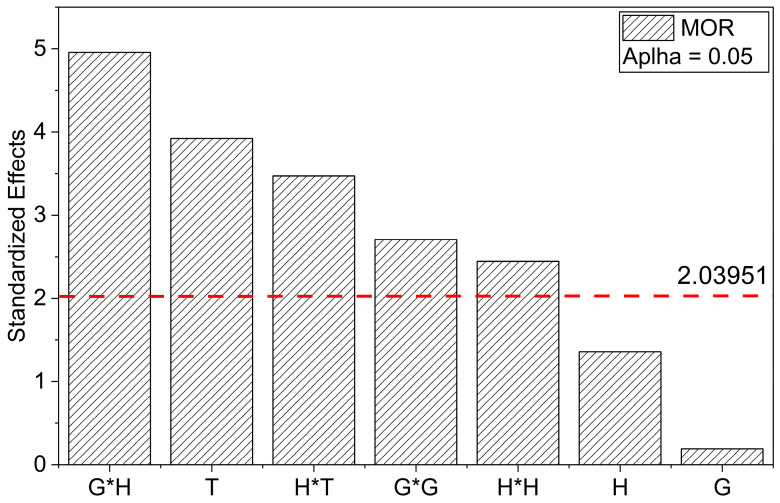
Pareto chart of standardized effects on the MOR at a 95% confidence level (α = 0.05). The red dashed line (t = 2.03951) indicates the significance threshold. The G*H interaction showed the strongest positive effect, followed by temperature (T) and H*T interaction, highlighting their major influence on MOR.

After identifying this formulation as an outlier, it was treated as a processing-induced anomaly in the MOR regression, and the model was re-fitted without this single point. The revised model showed improvements across all statistical indicators: R^2^ increased to 0.68, adjusted R^2^ to 0.60, and predicted R^2^ to 0.53, while the lack-of-fit became non-significant (*p* = 0.4124). Importantly, the hierarchy of significant effects remained unchanged, with MOR predominantly governed by the linear effect of T (*p* = 4.50 × 10^−4^) and the quadratic contributions of G^2^ (*p* = 0.0109) and H^2^ (*p* = 0.0204), indicating non-linear trends in nanofiller and hardener contents. From a physical perspective, the abnormally low MOR of the 2.0G/17H/30T specimen is consistent with the scenario of incomplete curing and microstructural defects (weak interfaces, microvoids, and GNP-rich agglomerates) expected under such conditions, as suggested by the inferior thermal performance of low-temperature-cured samples and by the more planar fracture regions and GNP clustering observed in non-optimized formulations. For transparency, the experimental MOR value of this condition is still reported in Table 4, but it is excluded from the final regression to avoid a single defective specimen dominating the response surface. The main mechanistic conclusions and the optimized formulation are therefore not affected by this treatment (Figure 3).

These findings strongly support the idea that removing a single inconsistent data point markedly enhanced both the statistical robustness and the interpretability of the model, revealing a more accurate picture of how GNP content, hardener proportion, and post-curing temperature synergistically influence flexural behavior. The refined model emphasizes the importance of multivariate optimization strategies for designing high-performance epoxy nanocomposites, where the full reinforcing potential of GNP can be realized only under carefully controlled conditions.

MOR (MPa) = 255.32 + 0.92G + 0.03H + 13.68T − 22.61G^2^ + 19.25H^2^ − 33.81 (G⋅H) − 32.06 (H⋅T) (4)

Beyond the statistical significance of T in the MOR model, these trends are consistent with the fracture mechanics of highly crosslinked epoxies. Post-curing at 120 °C increases crosslink density and reduces the fraction of unreacted epoxy and amine species, which would otherwise act as plasticizing domains and weak interfaces promoting microcrack nucleation under bending, in agreement with previous studies on the optimization of curing and post-curing conditions for improved toughness in epoxy systems [33]. Under these conditions, well-dispersed GNPs can effectively bridge microcracks and transfer load across the matrix–filler interface. In contrast, in under-cured networks their reinforcement action is partially masked by matrix yielding and interfacial debonding. Similar crack-bridging and load-transfer mechanisms have been reported for epoxy systems reinforced with functionalized graphene oxide and other 2D nanofillers, where an adequate curing schedule is essential to fully exploit the filler toughening potential [34].

In contrast, the statistical analysis of MOE revealed an excellent model fit, with R^2^ = 0.86, Adj. R^2^ = 0.84, and Pred. R^2^ = 0.82, confirming the high predictive power and reliability of the regression model. The low RMSE (0.571 GPa) indicates good agreement between the experimental and predicted values. The lack-of-fit was not significant (*p* = 0.483), indicating that the model adequately describes the experimental data without significant systematic error.

According to the ANOVA results, the linear terms G (*p* = 0.0305), H (*p* < 0.0001), T (*p* < 0.0001), and the interaction GH (*p* = 0.0015) significantly influenced MOE. T presented the highest contribution (47.32), followed by H (19.08), while G and GH also had significant but lower impacts. The main effects plots confirmed the negative influence of H and T, while G contributed positively, albeit to a lesser extent.

The regression model describing MOE as a function of the coded variables is given by:

MOE (GPa) = 10.32 + 0.26G − 0.89H − 1.40T − 0.57⋅G⋅H(5)

The negative coefficients for H, T, and G*H indicate that higher H content and higher T negatively affect MOE, while G shows a slight positive effect. The model confirms that lower H and T conditions favor higher stiffness, likely associated with reduced agglomeration and better dispersion [35]. The high R^2^ values and non-significant lack of fit validate the robustness of the model.

**Figure 4:**
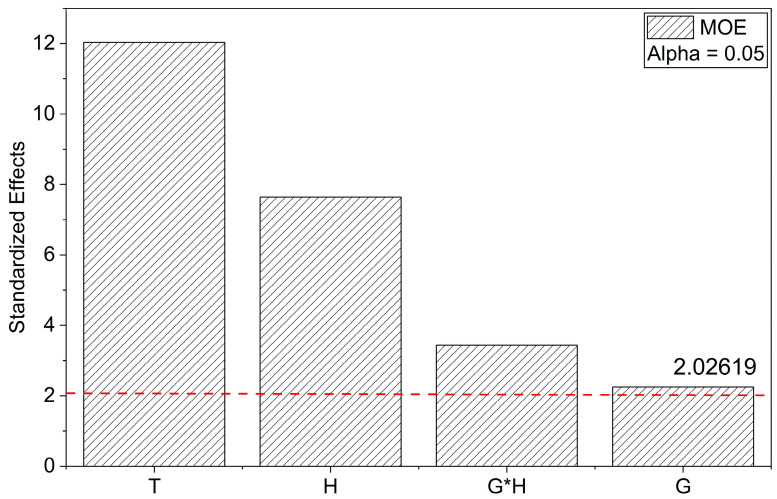
Pareto chart of standardized effects on the MOE at a 95% confidence level (α = 0.05). The red dashed line (t = 2.02619) indicates the significance threshold. T showed the strongest negative effect, followed by hardener content (H) and the H × T interaction, highlighting their major influence on MOE.

The influence of individual factors and their interactions on MOE was further elucidated through the Pareto chart and main effects plots. The Pareto chart (Figure 4) demonstrates that T and H exert the most pronounced negative effects on MOE, both exceeding the significance threshold at α = 0.05. The interaction term G*H also exhibited a statistically significant but comparatively smaller influence, whereas G contributed positively, albeit with a more moderate effect. These results corroborate the ANOVA’s statistical significance, confirming that increases in T and H lead to a substantial reduction in MOE, while G shows a slight improvement in stiffness.

**Figure 5:**
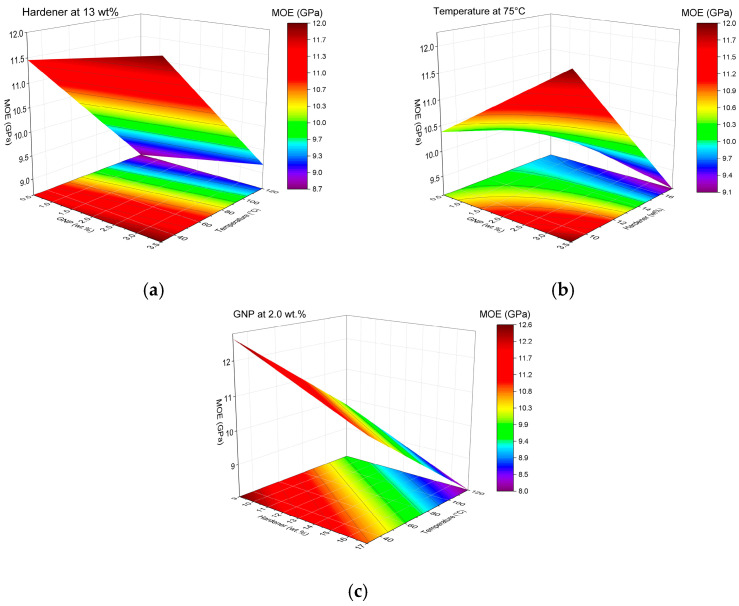
Response surface plots showing the combined effects of processing parameters on the MOE of GNP/epoxy nanocomposites: (**a**) GNP content and temperature at 13 wt.% hardener, (**b**) GNP content and hardener concentration at 75 °C, and (**c**) hardener concentration and temperature at 2.0 wt.% GNP.

The main effects plots reinforce these trends. As observed in Figure 4, MOE exhibits a marginal increase with increasing G, indicating a reinforcing effect of GNPs on the elastic modulus, though limited in magnitude. Figure 5 shows a pronounced reduction in MOE with increasing H, suggesting that higher H content may induce structural degradation, thereby reducing the material’s stiffness. Figure 5 further highlights the detrimental effect of T, with MOE decreasing sharply at elevated curing temperatures, likely due to excessive crosslink density or thermal degradation.

Overall, these graphical analyses are in complete agreement with the regression model and ANOVA results, emphasizing the predominant negative influence of H and T on MOE and the more modest, yet positive, contribution of G. These findings underscore the critical importance of optimizing H and T to preserve the elastic performance of GNP/epoxy nanocomposites.

In addition, the statistical analysis based on the BBD demonstrated that the reduced quadratic model for ε_f_ exhibited a high predictive capacity and excellent agreement with the experimental data. The coefficient of determination (R^2^) was 0.82, with an adjusted R^2^ of 0.80 and a predicted R^2^ of 0.77, indicating strong consistency between the fitted and predicted values and low residual dispersion (Root-MSE = 0.3418).

According to the ANOVA results, the curing temperature (T) was identified as the most statistically significant factor (*p* < 0.0001), exhibiting the highest F-value (161.76). This behavior was further supported by the coded coefficients, which showed a highly significant positive coefficient for T (+0.8875), indicating a strong positive effect of higher curing temperatures on the material’s deformability before fracture. Additionally, the interaction between G and H, represented by the G*H term, was also statistically significant (*p* = 0.0345) with a negative effect (−0.2167), suggesting that specific combinations of these parameters could negatively affect the strain-at-break behavior.

**Figure 6:**
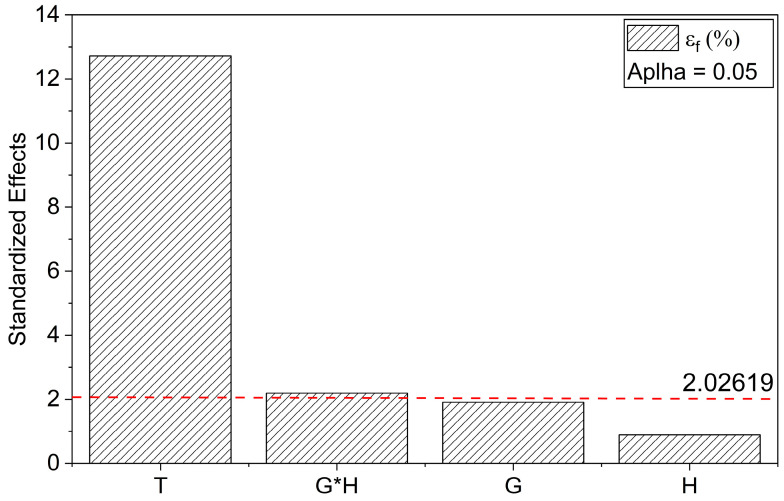
Pareto chart of standardized effects on the ε_f_ at a 95% confidence level (α = 0.05). The red dashed line (t = 2.02619) indicates the significance threshold. T showed the strongest positive effect, followed by the G × H interaction, highlighting their major influence on ε_f_.

The Pareto chart reinforces these findings by highlighting T as the predominant factor, largely exceeding the critical significance threshold (t = 2.026), while the G*H interaction also surpassed this threshold, confirming its relevance. In contrast, the individual effects of G and H were not statistically significant (*p* > 0.05), consistent with their low F-values (3.65 and 0.80, respectively) and their positions below the significance threshold in the Pareto plot (Figure 6).

The lack of significance for the main effect of G indicates that slight variations in GNP content alone do not substantially affect ε_f_ behavior, with its effect predominantly governed by its interaction with H. Although the lack-of-fit test was significant (*p* = 0.000357), potentially indicating limitations in the model’s ability to capture all experimental variability, the overall high R^2^ value and low PRESS (5.57) suggest robust predictive performance.

The final coded equation describing the flexural strain at break is expressed as:

ε_f_ = 2.99 − 0.13⋅G + 0.06⋅H + 0.89⋅T − 0.22⋅G⋅H (6)

This model unequivocally demonstrates that the optimization of flexural strain at break is predominantly driven by the curing temperature, with secondary but relevant contributions from the G*H interaction. Consequently, these parameters should be prioritized in future multivariable optimization strategies to maximize the mechanical performance of the studied nanocomposite system.

**Figure 7:**
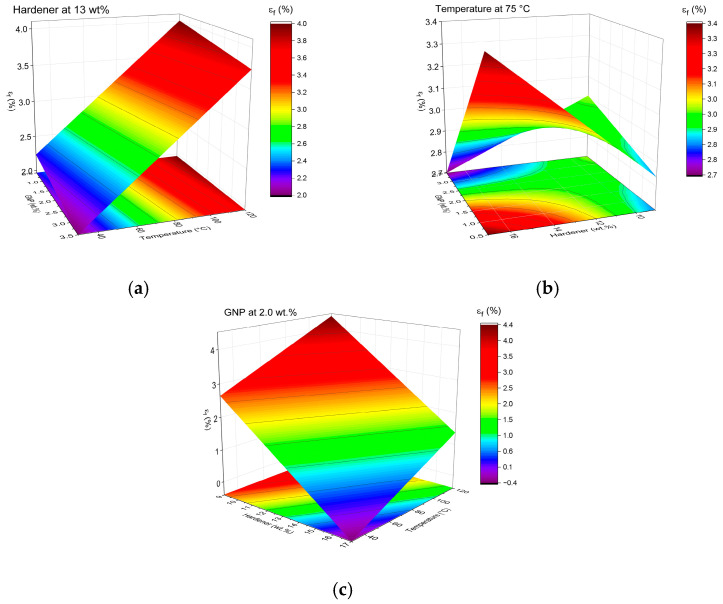
Response surface plots showing the combined effects of processing parameters on the ε_f_ of GNP/epoxy nanocomposites: (**a**) GNP content and temperature at 13 wt.% hardener, (**b**) GNP content and hardener concentration at 75 °C, and (**c**) hardener concentration and temperature at 2.0 wt.% GNP.

The three-dimensional response surface plots in Figure 7 provide additional insight into the influence of each independent variable on ε_f_, further substantiating the statistical findings. In the response surface associated with the G, a relatively flat profile was observed across the investigated concentration range, indicating a negligible effect on ε_f_ when G is varied independently. This is consistent with the non-significant *p*-value associated with G and reinforces the interpretation that the nanoparticle content alone does not meaningfully alter the strain-at-break response within the studied interval.

Similarly, the response surface for H shows modest curvature, with a slight peak at an intermediate level, suggesting an optimal range that mildly favors deformability. However, the overall effect remains statistically insignificant when considered in isolation, as previously demonstrated by the ANOVA results. This behavior may be attributed to the competing effects of increased crosslink density versus potential embrittlement at higher hardener concentrations.

In contrast, the surface plot of T shows an apparent, substantial increase in ε_f_ with increasing temperature, confirming the dominant role of this parameter in enhancing matrix ductility. This pronounced linear trend aligns with the large positive regression coefficient and the highly significant *p*-value (*p* < 0.0001), underscoring the thermal sensitivity of the network structure formed during post-curing. Elevated post-curing temperatures likely promote more effective crosslinking reactions and improved interfacial interactions between the matrix and nanofillers, ultimately increasing the material’s ability to accommodate deformation before fracture.

Taken together, these visual representations reinforce the conclusion that the post-curing temperature primarily governs ε_f_. At the same time, the negative interaction between G and H must be carefully managed to avoid detrimental combinations. The response surfaces thus serve as valuable tools for guiding the multivariable optimization of formulation and processing parameters, particularly in applications where maximizing toughness and flexibility is critical.

To provide a more comprehensive assessment of the mechanical performance achieved in this study, the flexural properties of the optimized GNP/epoxy nanocomposite were benchmarked against those reported in the literature for both neat DGEBA/TETA epoxy and epoxy composites reinforced with graphene oxide (GO) or natural fibers. Table 5 summarizes this comparison, presenting the MOR and MOE, along with the respective percentage improvements obtained by the optimized formulation developed herein.

The flexural performance of the optimized GNP nanocomposite (2.0G/9H/120T) was benchmarked against neat DGEBA/TETA epoxy and representative systems reinforced with natural fibers and graphene derivatives reported in the literature (Table 5). The optimized formulation achieved a MOR of 322 MPa and a MOE of 9.7 GPa, representing exceptional improvements relative to the neat epoxy and surpassing the performance of most reported nanofiller systems.

Compared to neat DGEBA/TETA epoxy (MOR ≈ 70–110 MPa; MOE ≈ 2.2–3.0 GPa [36,37,38]), the optimized composite exhibited increases of +258% in MOR and +273% in MOE, confirming a strong reinforcing effect driven by both the GNPs and the statistically optimized curing parameters. These enhancements are attributed to (i) the uniform nanoscale dispersion of GNPs, (ii) improved interfacial adhesion via π–π interactions between the graphene surface and the aromatic epoxy network, and (iii) higher crosslink density resulting from the post-curing at 120 °C.

Relative to GO- and rGO-reinforced systems (MOR ≈ 98–150 MPa; MOE ≈ 3.2–3.6 GPa [38]), the optimized nanocomposite achieved gains of +115–247% in strength and +166–203% in stiffness. This corroborates the consensus that pristine or lightly oxidized graphene structures, such as GNPs, outperform GO/rGO in flexural loading due to preservation of the sp^2^ lattice, enabling efficient stress transfer without the structural defects introduced by oxidation [46,47]. GO and rGO, while facilitating chemical bonding via oxygenated groups, disrupt the conjugated network, thereby reducing the nanoscale load-bearing efficiency.

For graphite/epoxy systems (MOR ≈ 80–90 MPa; MOE ≈ 3.0 GPa [41]), relative improvements reached +279% and +213%, respectively, underscoring that poor exfoliation and low interfacial activity limit reinforcement efficiency when compared with exfoliated nanostructures. Similarly, pristine graphene/epoxy composites (MOR ≈ 119 MPa; MOE ≈ 3.4 GPa [42]) displayed more modest gains (ΔMOR ≈ +171%; ΔMOE ≈ +185%), reflecting the importance of preventing nanosheet aggregation during processing.

The inclusion of amine-functionalized graphene (NH_2_-GNs) has been reported to further improve interfacial bonding through covalent interactions between amino groups and epoxy chains, yielding MOR ≈ 152 MPa and MOE ≈ 5.4 GPa [46]. Nevertheless, even with such chemical compatibilization, the present GNP system achieved more than twice the strength and nearly double the stiffness, confirming the superior reinforcing potential of well-dispersed multilayer nanoplatelets under optimized curing conditions.

Remarkably, the 3D graphene foam/epoxy composite (≈0.6 vol.% foam, <0.1 wt.%) reported by Jia et al. [47] reached MOR ≈ 141 MPa and MOE ≈ 5.3 GPa, with improvements of +68% in strength and +90% in stiffness over neat epoxy, values still significantly below those of the optimized GNP composite. Despite the continuous load-bearing network provided by the graphene skeleton, the foam’s volumetric constraint and its limited interfacial area limit overall reinforcement compared to the uniformly dispersed GNP system.

When compared with natural-fiber composites, the magnitude of improvement becomes even more evident. Composites reinforced with date-palm or jute fibers typically show MOR ≈ 30–230 MPa and MOE ≈ 3–6 GPa [32,39], while NaOH/GO-modified bamboo fiber systems can reach MOR ≈ 335 MPa and MOE ≈ 23.8 GPa [40]. Although the latter presents comparable flexural strength (ΔMOR ≈ +8%) and higher stiffness (ΔMOE ≈ −52%) relative to the present nanocomposite, these macroscale systems rely on high fiber contents (≈40–50 vol.%) and exhibit strong anisotropy. Conversely, the GNP/epoxy composite achieves similar or superior strength with only 2 wt.% filler, while maintaining isotropy and simple processing.

The overall flexural analysis confirms a consistent trend: the optimized GNP/epoxy formulation surpasses conventional GO, rGO, graphite, and pristine graphene systems, as well as fiber-reinforced composites, while requiring minimal filler content and simpler processing [48,49,50]. In summary, the optimized 2.0G/9H/120T nanocomposite establishes a new benchmark in DGEBA/TETA systems, achieving >250% increase in flexural strength and >270% in stiffness over neat epoxy, and outperforms previously reported GO, rGO, graphite, NH_2_-GNs, and graphene-foam composites. These results confirm that precise control of filler dispersion and curing parameters enables flexural performance comparable to, or exceeding, that of hybrid fiber composites, while ensuring superior uniformity and scalability.

### 3.3. Viscoelastic Behavior of the GNP-Reinforced Epoxy Nanocomposites

**Figure 8:**
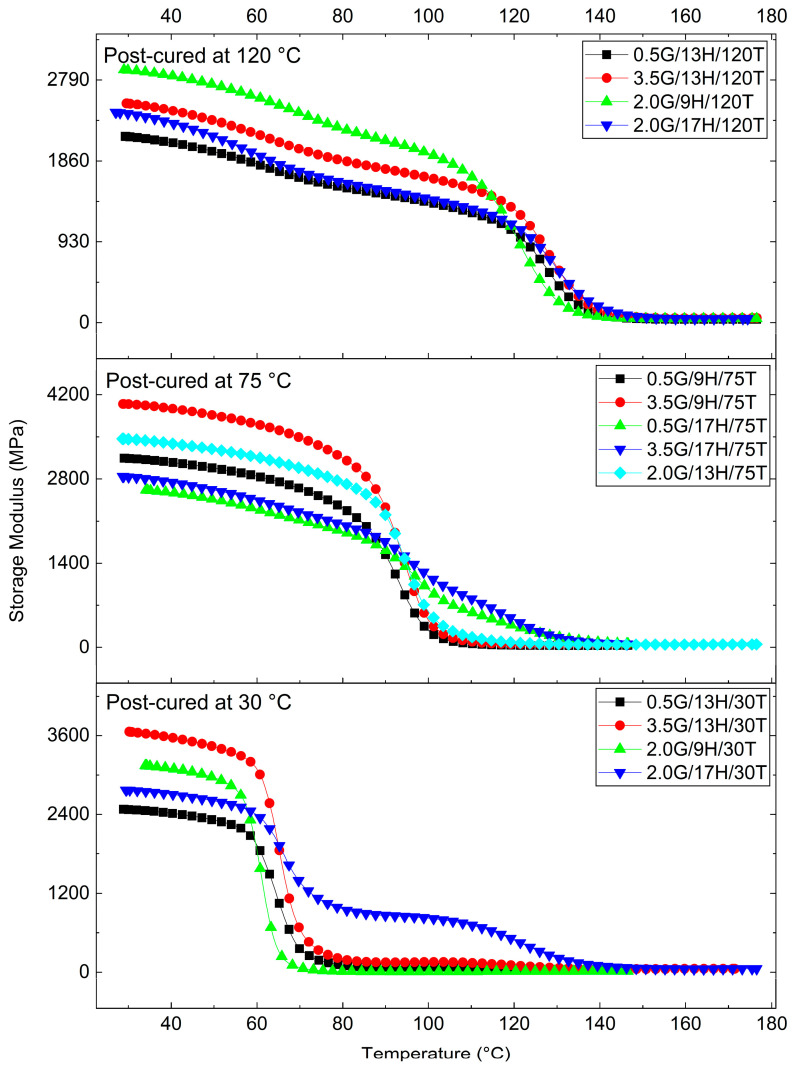
Temperature dependence of the E′ for GNP-reinforced epoxy nanocomposites post-cured at 30, 75, and 120 °C.

**Figure 9:**
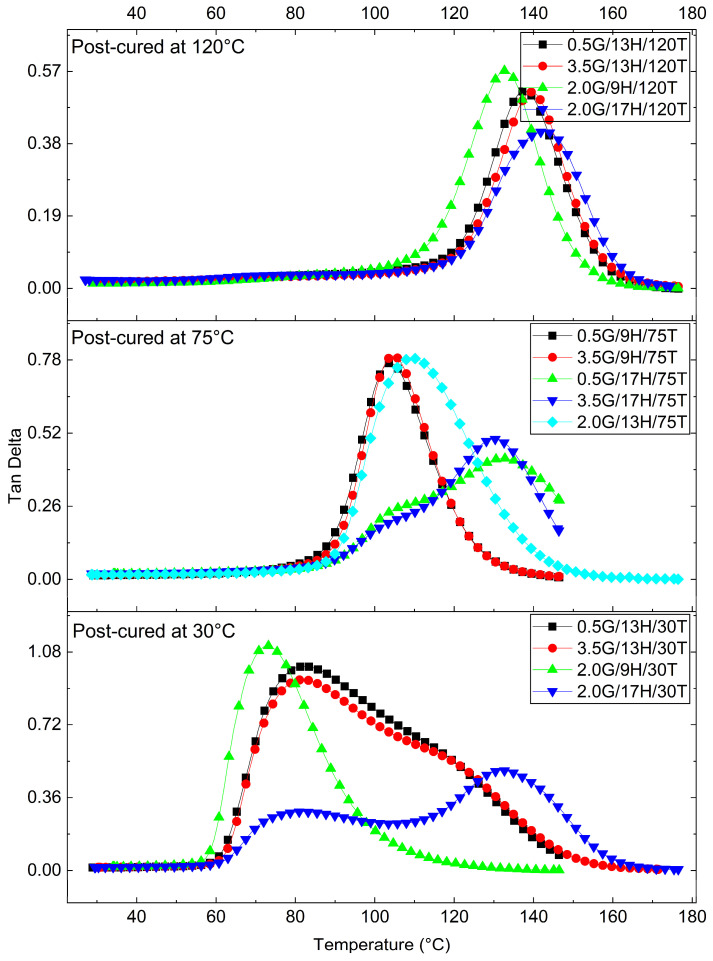
Temperature dependence of the tanδ for GNP-reinforced epoxy nanocomposites post-cured at 30, 75, and 120 °C.

**Figure 10:**
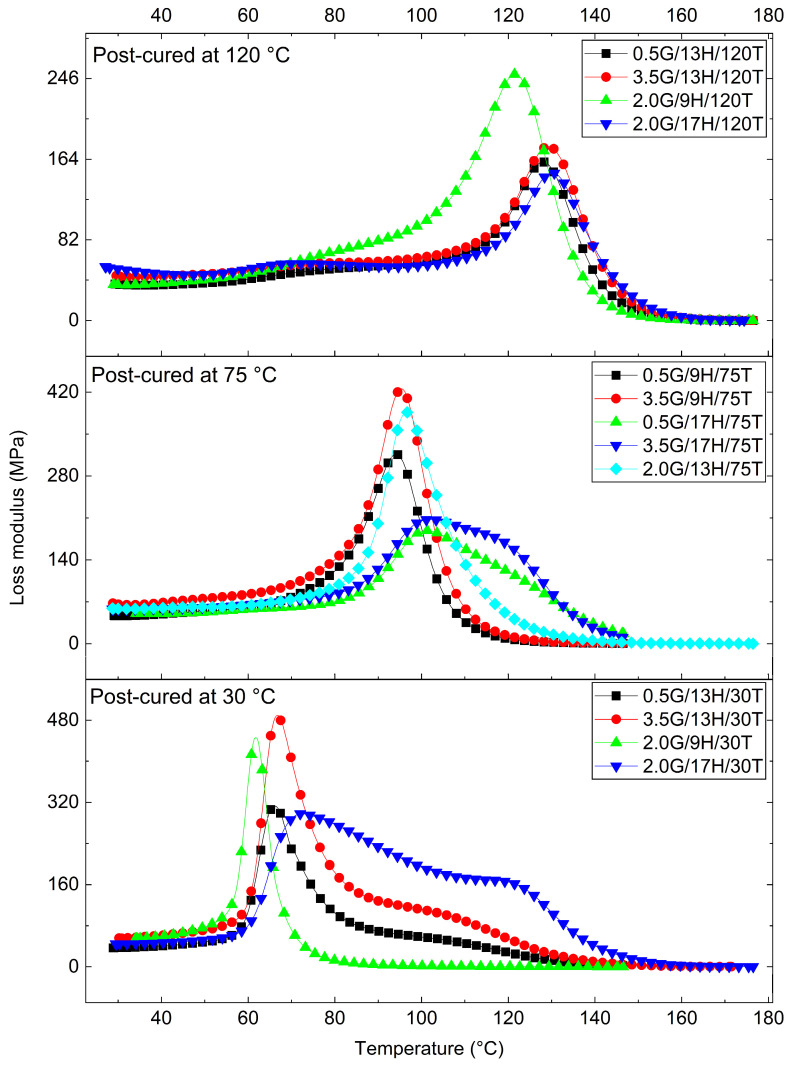
Temperature dependence of the E″ for GNP-reinforced epoxy nanocomposites post-cured at 30, 75, and 120 °C.

DMA was performed to elucidate the viscoelastic response of the GNP/epoxy nanocomposites. From the temperature-dependent curves of the storage modulus (E′), loss modulus (E″), and damping factor (tanδ), key viscoelastic parameters were extracted: the glass transition temperature (Tg) (from the tanδ peak), the storage moduli in the glassy [E′ (30 °C)] and rubbery [E′ (Tg + 30 °C)] states, the logarithmic drop (ΔlogE′) between these regions, the maximum damping factor (tanδ_max_), and the crosslink density (ν_e_) estimated by the rubber elasticity model [51]. The main results are summarized in Table 6. At the same time, Figure 8, Figure 9 and Figure 10 depict the evolution of E′, E″, and tanδ, respectively, grouped by post-curing temperature (30, 75, and 120 °C), which was identified as the most influential factor governing the dynamic mechanical response.

The results reveal a strong dependence of viscoelastic properties on curing conditions and GNP content. The Tg ranged from 72.9 °C to 143.0 °C, reflecting the synergistic effect of G, H, and T. Samples post-cured at 120 °C exhibited the highest Tg values (≥130 °C), indicative of a highly crosslinked, rigid polymeric network with restricted chain mobility. Conversely, the specimens cured at 30 °C displayed Tg below 85 °C, consistent with incomplete network formation and residual unreacted epoxy [51,52]. The intermediate group, cured at 75 °C, showed Tg values of 95–116 °C, corresponding to partially cured, moderately crosslinked matrices [53,54].

The storage modulus in the glassy region [E′ (30 °C)], ranging from 2.1 to 4.0 GPa, confirms the high stiffness of the epoxy matrix and the reinforcing effect of GNPs at small deformations. As the temperature increased beyond Tg, E′ dropped sharply to 9–137 MPa, defining the rubbery plateau [53]. The ΔlogE′ values, varying from 1.4 to 2.5, correspond to a two-to-three-order-of-magnitude decrease, typical of well-crosslinked thermosets. Significantly, the presence of GNP maintained residual stiffness above Tg, suggesting efficient load transfer at the filler–matrix interface and enhanced interfacial adhesion [55,56].

These tendencies are clearly observable in Figure 8, where the modulus–temperature curves exhibit progressively higher stiffness and delayed softening with increasing post-curing temperature. In particular, the samples post-cured at 120 °C maintain elevated E′ up to approximately 120 °C before the α-transition. In contrast, those cured at 30 °C show an early modulus drop near 60–80 °C, evidencing incomplete cure and weaker network integrity.

The tanδ curves (Figure 9) corroborate these findings, providing direct evidence of how post-curing and composition influence relaxation dynamics. Samples cured at 30 °C exhibited broad and asymmetric peaks (tanδ_max_ ≈ 1.0–1.1), characteristic of network heterogeneity and a broad relaxation-time distribution. In contrast, post-curing at 120 °C produced narrow, well-defined peaks (tanδ_max_ ≈ 0.4–0.6), shifted to higher temperatures, confirming the development of a homogeneous, densely crosslinked network. The H also affected the peak morphology: increasing H from 9 to 17 phr broadened the tanδ band and slightly shifted it toward higher temperatures, indicating localized stoichiometric imbalance and microstructural heterogeneity. Additionally, GNP incorporation reduced the peak intensity, reflecting its role as a mechanical constraint that limits polymer segmental motion and energy dissipation, thereby enhancing elastic storage. Although the exfoliation state and interfacial chemistry were not quantified in detail, the formulations that combine higher E′ and lower tanδ_max_ in Figure 8, Figure 9 and Figure 10 correspond to those in which the SEM analysis reveals more homogeneous GNP dispersion with fewer large agglomerates. This suggests that better-exfoliated, high-aspect-ratio nanoplatelets and stronger interfacial bonding promote more efficient load transfer and the formation of constrained interphase regions, which jointly contribute to the observed stiffening and reduced damping.

The H also affected the peak morphology: increasing H from 9 to 17 phr broadened the tanδ band and slightly shifted it toward higher temperatures, indicating localized stoichiometric imbalance and microstructural heterogeneity. Additionally, GNP incorporation reduced the peak intensity, reflecting its role as a mechanical constraint that limits polymer segmental motion and energy dissipation, thereby enhancing elastic storage.

The E″ curves (Figure 10) showed similar trends, with higher, narrower peaks for the post-cured samples, supporting the correlation between improved crosslinking and more concentrated relaxation processes. The magnitude and temperature of the E″ maxima increased systematically with post-curing, demonstrating the reinforcement of the relaxation mechanism and the delayed onset of viscoelastic flow in the thermoset network.

The crosslink density (ν_e_), calculated from the rubbery modulus as:

ν_e_ = (E′_rub_)/3RT (7)

ranged from 3.2 × 10^−3^ to 14.2 × 10^−3^ mol·m^−3^ (Table 6). Higher ν_e_ values were obtained for the formulations post-cured at 120 °C and for samples containing well-dispersed GNPs, such as 3.5G/13H/30T and 0.5G/13H/120T, confirming that thermal activation enhances chemical crosslinking, while the nanofiller induces additional physical crosslinks by restricting polymer chain motion through strong interfacial interactions. Lower ν_e_ values observed in samples such as 0.5G/13H/30T and 2.0G/9H/30T are consistent with lower Tg and broader damping peaks, reflecting less-integrated network structures [51].

A comparative analysis of all formulations demonstrates that T is the dominant parameter controlling viscoelastic behavior, followed by H and G. Increasing T results in higher Tg, greater E′, lower tanδ_max_, and higher ν_e_, confirming the formation of more cohesive, thermally stable networks. While higher H levels (17 phr) slightly enhanced stiffness, they also broadened the relaxation transitions, suggesting that excessive hardener may create local over-crosslinked regions or unreacted residues.

The addition of GNP consistently increased E′ (30 °C) and decreased tanδ_max_, confirming its role in reinforcing the matrix and improving the elastic recovery. The most rigid and thermally stable nanocomposites were those post-cured at 120 °C, particularly 3.5G/13H/120T and 2.0G/17H/30T, which combined high Tg and stiffness with moderate ΔlogE′ and low damping. Conversely, the least rigid systems, such as 0.5G/13H/30T and 2.0G/9H/30T, displayed lower Tg and higher damping, typical of under-cured or poorly interconnected networks [52,53,54,55].

Overall, the DMA results demonstrate that the combined effects of GNP content, hardener concentration, and post-curing temperature enable precise tailoring of the viscoelastic performance of epoxy nanocomposites. The interplay between chemical crosslinking (driven by curing kinetics) and physical reinforcement (induced by GNP interfacial constraints) governs both the stiffness and damping behavior of the hybrid network. The consistent evolution of Tg, E′, tanδ, and ν_e_ confirms that the selected processing parameters are sufficient to tune the balance between elastic energy storage and viscous energy dissipation in these multifunctional epoxy systems.

Although cure kinetics were not directly quantified in this study, the combined evolution of Tg, ν_e_, ΔlogE′, and tanδ strongly suggests that the main effect of the GNPs is to restrict the segmental mobility of an already-formed network rather than to drastically change the global activation energy of curing. The decrease in tanδ_max_ and the narrowing of the relaxation peaks with increasing T and moderate GNP loadings indicate a transition from heterogeneous, under-cured networks to more uniform structures in which the nanoplatelets topologically constrain polymer chains. This mechanistic picture is consistent with literature reports on epoxy systems modified with GO and MoS_2_, where 2D fillers predominantly introduce physical crosslinks and interfacial friction that slow segmental dynamics, while only modestly affecting the overall curing kinetics [55].

In the following subsection, the BBD and RSM are applied to quantitatively assess the main and interaction effects of G, H, and T on E′ (30 °C) and tanδ_max_, enabling the establishment of predictive models that correlate processing conditions with the viscoelastic behavior of GNP-based epoxy nanocomposites.

#### Response Surface Analysis of Dynamic Mechanical Parameters (E′ and tanδ)

The viscoelastic behavior of the GNP/epoxy nanocomposites was statistically optimized using a BBD, with G, H, and T defined as independent factors. The responses selected from DMA were the E′ and the tanδ_max_, representing, respectively, the elastic and viscous contributions to the composite behavior.

Effect of G, H, and T on E′ (30 °C)

The RSM based on a BBD was applied to evaluate the effects of G, H, and T on the E′ of the epoxy nanocomposites. The quadratic model was found to be statistically significant, with determination coefficients R^2^ = 0.83, R^2^adj = 0.77, and R^2^pred = 0.59, confirming its adequacy for predictive purposes. The coded regression equation for E′ (MPa) was obtained as:

E’ = 3289.51 + 336.36 G − 326.48 H − 259.99 T − 537.58 T^2^ (8)

The ANOVA results demonstrated that all linear terms (G, H, and T) and the quadratic term T^2^ were statistically significant (*p* < 0.05), confirming that both compositional and thermal variables exert substantial influence on the stiffness of the cured network. The absence of significant interaction terms suggests that each factor primarily affects the modulus independently [56].

**Figure 11:**
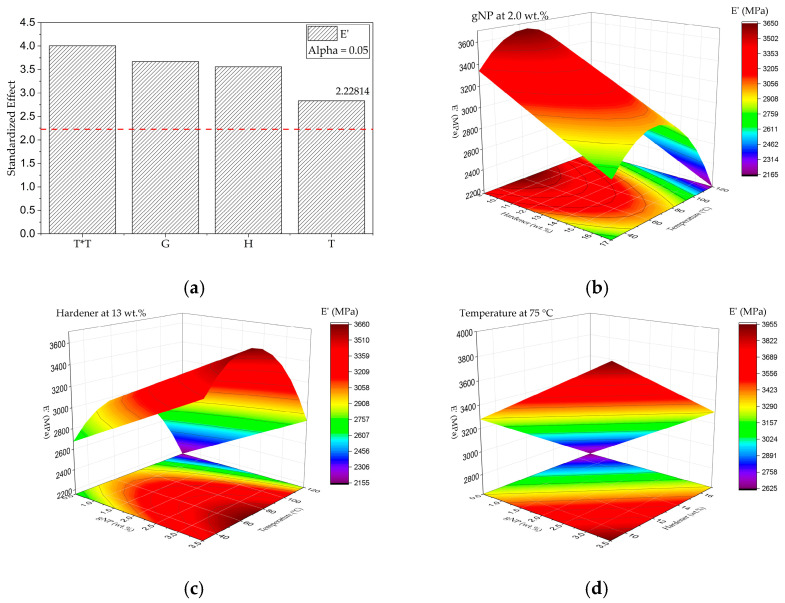
BBD results for E’: (**a**) Pareto chart (the red line represents the threshold for statistical significance of the effects); (**b**) Response surface plot with G at 2.0 wt.%; (**c**) Response surface plot with H at 13 wt.%; (**d**) Response surface plot with T at 75 °C.

The Pareto chart (Figure 11a) further supports these observations, revealing that the quadratic temperature term (T^2^) exerts the most substantial standardized effect, followed by the positive contribution of G and the negative influences of H and T. This pattern emphasizes that while higher post-curing temperatures initially enhance stiffness through increased crosslinking, excessive thermal exposure (beyond ~100 °C) may cause partial chain relaxation or localized over-curing, reducing E′.

The main effects plots and response surfaces (Figure 11b–d) clearly illustrate these tendencies. The E′ increases nearly linearly with the graphene nanoplatelet concentration up to 3.5 wt.%, indicating effective load transfer through strong interfacial adhesion and homogeneous GNPs dispersion in the matrix. Conversely, higher hardener content (above 13 phr) slightly decreases E′, possibly due to a stoichiometric imbalance that leads to unreacted amine domains and network irregularities [57,58].

Regarding temperature, a non-linear behavior was observed: E′ rises steadily from 30 °C to approximately 75–90 °C, associated with enhanced cure conversion and increased crosslink density, but decreases at 120 °C, evidencing the curvature predicted by the significant T^2^ term. This drop is consistent with post-curing-induced molecular relaxation and possible reduction in secondary interactions at excessive thermal energy [59,60].

The trends observed for E′ align with those from DSC and TGA analyses, in which higher curing temperatures (up to 120 °C) yielded higher Tg and thermal stability, confirming the completion of crosslinking reactions. However, the mechanical relaxation at extreme conditions rationalizes the slight reduction in E′ at the highest T, reflecting a transition from an optimal to an over-cured state.

The same relationship was corroborated by the flexural results, where the compositions with intermediate-to-high post-curing temperatures exhibited the highest modulus and strength values, paralleling the dynamic stiffness behavior. In particular, the 2.0G/9H/120T sample achieved the best overall performance, combining a high storage modulus, a high Tg (~140 °C), and superior static mechanical properties.

Overall, the RSM analysis confirmed that E′ is predominantly governed by both physical reinforcement from GNP and chemical crosslinking controlled by temperature and hardener ratio. The curvature effect of T^2^ underscores the existence of an optimal thermal window for achieving maximum stiffness without degradation of molecular order. The model’s statistical robustness (R^2^adj > 0.75) and the physical consistency across DMA, DSC, TGA, and flexural results validate the predictive power of the proposed response surface for tailoring viscoelastic performance in GNP/epoxy nanocomposites.

Effect of G, H, and T on Tg (DMA)

The damping behavior of the GNP/epoxy nanocomposites, as reflected in the maximum temperature on the tanδ curve, was analyzed using BBD to elucidate how H and T affect the dynamic glass-transition response of the polymeric matrix. The quadratic regression model was statistically significant, presenting R^2^ = 0.88, R^2^adj = 0.85, and R^2^pred = 0.57, with low residual variance (Root-MSE = 8.67). These values indicate an adequate fit and strong predictive capability. The coded equation derived from the analysis was:

Tg (DMA) (°C) = 112.07 + 12.75 H + 23.10 T − 12.28 (HT)(9)

**Figure 12:**
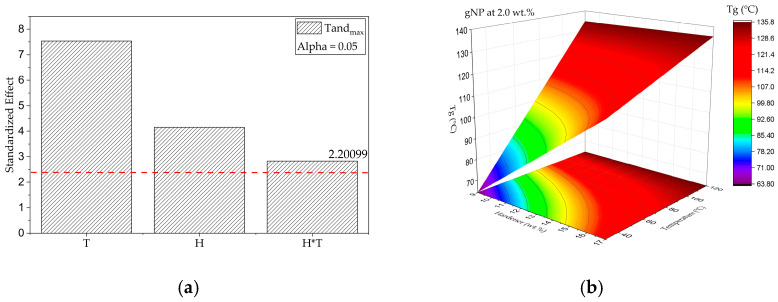
BBD results for tanδ_max_: (**a**) Pareto chart (the red line represents the threshold for statistical significance of the effects); (**b**) Response surface plot with G at 2.0 wt.%.

The ANOVA results revealed that both H (*p* = 0.0016) and T (*p* < 0.0001) significantly influence tanδ_max_, and that the interaction term (H × T) also had a meaningful effect (*p* = 0.016). The Pareto chart (Figure 12a) corroborates these findings, ranking T as the dominant factor, followed by H, and finally their negative interaction (H × T). This indicates that increasing either temperature or hardener content individually enhances molecular mobility and energy dissipation, whereas simultaneous increases reduce damping due to enhanced crosslinking efficiency and restricted segmental motion [46].

The response surface plot (Figure 12b) confirms that tanδ_max_ initially rises with increasing curing temperature, reaching a maximum near intermediate levels (≈75–90 °C), where chain mobility is sufficient to dissipate energy. Beyond this range, however, tanδ_max_ decreases sharply, consistent with the formation of a denser and more homogeneous crosslinked structure that limits molecular relaxation. Likewise, higher hardener ratios promote a more complete curing reaction but may cause local stoichiometric imbalances at excessive concentrations, resulting in broader relaxation spectra and slightly higher damping [33].

The lack-of-fit test was statistically significant (F = 15.95; *p* = 0.00207), suggesting that the current quadratic model does not fully capture some additional nonlinearities. Nonetheless, the model remains reliable given its high R^2^ and R^2^adj values, small residual variance (Root-MSE = 8.67), and excellent physical consistency with the experimental DMA, DSC, and flexural data. The observed lack of fit likely arises from complex curing kinetics and nanoscale interactions between the GNP fillers and the polymer matrix, which introduce additional relaxation heterogeneity beyond simple quadratic behavior.

The statistical trends identified here align closely with the DMA curves previously discussed. Samples post-cured at lower temperatures exhibited broader and higher tanδ peaks (≈1.0–1.1), characteristic of heterogeneous and partially cured networks, whereas those cured at 120 °C presented narrower and lower peaks (≈0.4–0.5), consistent with more uniform crosslink distributions. This inverse correlation between E′ and tanδ_max_ reflects the intrinsic balance between elastic storage and viscous dissipation, a hallmark of densely crosslinked thermoset systems reinforced with rigid nanoparticles [33].

**Figure 13:**
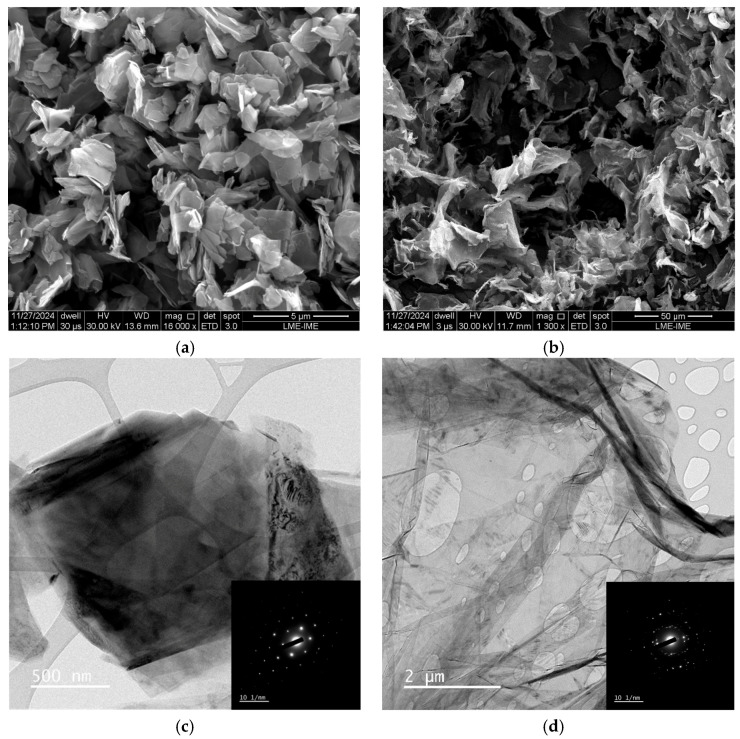

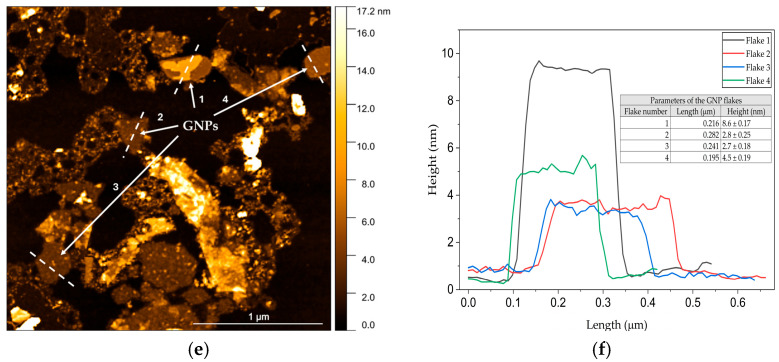
Morphological and structural characterization of graphite and GNPs: (**a**) SEM image of graphite, showing compact micrometric flakes with layered structure; (**b**) SEM image of GNPs, showing exfoliated and wrinkled thin sheets; (**c**) TEM image of a graphite flake with SAED pattern (inset) showing sharp diffraction spots, typical of a single crystal; (**d**) TEM image of GNPs with SAED pattern (inset) displaying concentric rings of discrete spots, indicating a polycrystalline structure; (**e**) AFM topography; and (**f**) corresponding height profiles of GNPs. The analyzed flakes (1–4) exhibit lateral sizes of 0.19–0.28 µm and thicknesses of 2.7–8.6 nm, indicating a few-layer graphene morphology with partial restacking.

In addition, the nanoscale morphology of the GNPs helps rationalize the observed damping behavior. The AFM and TEM analyses (Figure 13) show few-layer, high-aspect-ratio platelets with partial restacking, which are expected to create an extended interphase where chain mobility is constrained under oscillatory loading. This constrained interphase efficiently stores elastic energy (higher E′). It reduces viscous dissipation (lower stanδ_max_) at intermediate GNP contents, whereas excessive agglomeration can locally decouple the filler from the matrix and partially restore energy dissipation.

Furthermore, these results corroborate the DSC and TGA analyses, which showed increasing Tg and thermal stability with higher curing temperature and optimized H ratio. The 2.0G/9H/120T composition again emerged as the optimized formulation, exhibiting the highest Tg (~143 °C), lowest tanδ_max_, and superior stiffness, confirming its well-developed network structure and effective interfacial load transfer.

In summary, the BBD analysis demonstrated that the post-curing temperature primarily governs tanδ_max_, while the hardener ratio modulates this effect through its interaction with it. The significant negative H × T term reflects the synergistic curing behavior that minimizes energy dissipation as the system transitions from under-cured to optimally crosslinked. Together with the E′ model, these findings highlight that controlling the interplay between chemical crosslinking and nanoparticle reinforcement enables precise tailoring of the viscoelastic performance of the GNP/epoxy nanocomposites.

### 3.4. Morphological Characterization

The SEM micrograph of pristine graphite (Figure 13a) reveals large, compact micrometric flakes with a well-defined lamellar morphology. The platelets exhibit smooth surfaces and a stacked arrangement, typical of highly ordered graphitic structures, which limit surface area exposure. In contrast, the SEM image of the graphene nanoplatelets (Figure 13b) shows extensive exfoliation, with individual sheets partially separated from the bulk structure and displaying irregular, wrinkled morphologies. These wrinkles and folds increase the accessible surface area and indicate a reduction in the number of stacked layers, a feature that enhances interfacial interactions when incorporated into polymer matrices. Furthermore, the increased roughness and apparent porosity observed in the GNP sample suggest a more open structure, which can facilitate mechanical interlocking and stress transfer within nanocomposites [61].

TEM analysis provides complementary nanoscale evidence of these morphological changes. The TEM image of graphite (Figure 13c) confirms the presence of thick, multilayered stacks with well-ordered crystalline domains, as evidenced by the sharp and discrete diffraction spots in the SAED pattern, characteristic of a single-crystal-like arrangement. In contrast, the GNP sample (Figure 13d) exhibits thinner, more transparent nanosheets with overlapping regions and a lower degree of stacking, consistent with partial exfoliation into few-layer graphene. The corresponding SAED pattern displays concentric rings composed of discrete spots, indicative of a polycrystalline nature and smaller crystalline domains. This transition from compact, thick graphite flakes to thin, wrinkled, and partially transparent graphene nanoplatelets confirms the successful exfoliation process, yielding a more favorable morphology for dispersion and load transfer in epoxy nanocomposites [61].

The AFM topographic image (Figure 13e) reveals the surface morphology of the GNPs, showing irregularly shaped flakes with lateral dimensions of approximately 0.19–0.28 µm. The corresponding height profiles (Figure 13f) indicate thicknesses of 2.7 ± 0.18 nm to 8.6 ± 0.17 nm, values characteristic of few-layer graphene. The image also exhibits subtle drying marks and surface irregularities, which can be attributed to the sample preparation process, particularly to solvent evaporation during deposition. Despite these artifacts, the GNP flakes are clearly discernible, allowing reliable evaluation of their lateral size and thickness. The thinner regions (2–3 nm) correspond to well-exfoliated platelets composed of a few graphene layers, whereas thicker domains (>5 nm) likely result from partial restacking or local overlapping of multiple flakes. This morphological heterogeneity is consistent with previous studies, where Wei et al. [12] reported similar height ranges (2–10 nm) for exfoliated GNPs dispersed in epoxy matrices, and Agustina et al. [13] demonstrated that platelet size and aggregation degree critically influence mechanical reinforcement and thermal stability. Such features highlight the typical morphology of solution-processed GNPs, where restacking during drying is unavoidable but still compatible with effective interfacial contact when incorporated into polymer matrices. Overall, the AFM results confirm the presence of few-layer graphene flakes with moderate stacking, which are suitable for enhancing the mechanical and thermal performance of epoxy nanocomposites.

From a mechanical standpoint, this combination of sub-micrometric lateral dimensions and few-layer thickness implies relatively high aspect ratios and significant interfacial area available for stress transfer. In our previous work on the same GNP grade, a size distribution obtained from SEM image analysis revealed a broad population of platelets with lengths spanning the sub-micrometre to tens-of-micrometre range, fitted by a Weibull distribution and confirming a heterogeneous but predominantly high-aspect-ratio morphology [62]. Together with the partial exfoliation evidenced by TEM/AFM, this morphology is consistent with the enhanced flexural stiffness and reduced damping observed here, as elongated platelets and their interphase regions effectively bridge microcracks and restrict local chain relaxation during bending and dynamic loading.

**Figure 14:**
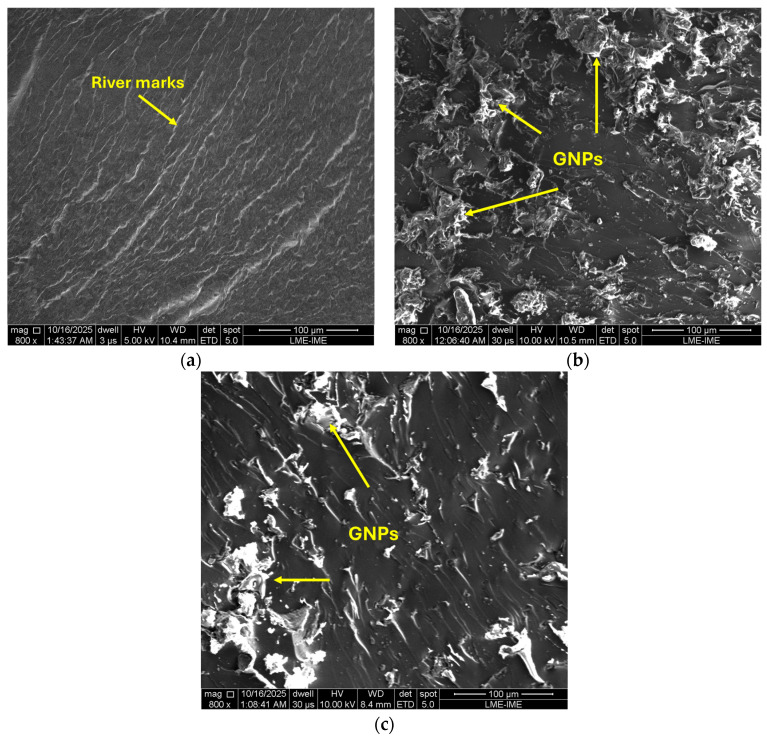
SEM micrographs of the fracture surfaces of selected nanocomposites: (**a**) plain epoxy, (**b**) 3.5G/9H/75T, (**c**) 2.0G/9H/120T. The yellow arrows indicate agglomerates of GNP on the fracture surfaces, highlighting regions of particle clustering that influence the composite morphology.

Figure 14 presents representative SEM micrographs of the fracture surfaces for (a) neat epoxy, (b) 3.5G/9H/75T, and (c) the optimized formulation 2.0G/9H/120T. The neat epoxy (Figure 14a) exhibits characteristic river marks and relatively smooth planes typical of brittle fracture in crosslinked DGEBA/TETA systems. These features indicate unstable crack propagation with limited plastic deformation, consistent with the low strain-to-failure and relatively low fracture toughness commonly reported for neat epoxy resins [63,64].

In contrast, the sample with a higher graphene nanoplatelet content (3.5 wt.%) (Figure 14b) reveals a significantly rougher and more heterogeneous fracture morphology, characterized by large GNP agglomerates and distinct platelet edges embedded within the polymer matrix. This morphology evidences a transition from brittle to quasi-ductile fracture, in which the crack front is repeatedly deflected and branched due to the rigid two-dimensional nanofillers. The increased surface roughness and the appearance of secondary crack paths denote enhanced energy dissipation through mechanisms such as crack deflection, interfacial debonding, and graphene pull-out, as widely reported for GNP-reinforced epoxy systems [65,66,67]. However, excessive filler content leads to pronounced agglomeration and stress concentration, which may locally compromise load transfer and fracture uniformity [68].

The optimized nanocomposite (2.0G/9H/120T) (Figure 14c) exhibits a more homogeneous dispersion of GNPs, with fewer and smaller clusters compared to the 3.5 wt.% formulation. The fracture surface is characterized by multiple fracture planes, microvoid coalescence, and tortuous crack paths, indicating efficient interfacial bonding and effective stress transfer. These features are consistent with the superior flexural strength and modulus, as well as the increased E′ and Tg determined by DMA and DSC, respectively. The coexistence of deflection, pinning, and microplastic deformation around GNPs provides evidence of a hybrid toughening mechanism, as also observed in other graphene/epoxy systems [69,70,71]. These morphological observations are in good agreement with the optimized viscoelastic response discussed in Section 3.3, indicating that GNP morphology, degree of exfoliation, and interfacial bonding jointly govern crack bridging and load-transfer efficiency under flexural loading, as well as the development of constrained interphase regions that influence the damping behavior.

From a mechanistic standpoint, the addition of GNPs modifies the fracture behavior through several synergistic processes:

- Crack deflection and branching, which increase the effective fracture surface area;
- Graphene pull-out and interfacial debonding, absorbing additional fracture energy;
- Plastic deformation in the epoxy matrix adjacent to graphene platelets, induced by strong interfacial adhesion;
- Crack pinning, where nanoplatelets act as barriers to crack propagation, creating tortuous crack paths and enhancing resistance to crack growth.

Such toughening mechanisms are widely reported in the literature. Yao et al. [67] and Chong et al. [68] observed similar roughened and multi-planar fracture morphologies in epoxy systems reinforced with hybrid GNP/CNT nanofillers, whereas Khoramishad et al. [69] described comparable crack deviation and pull-out effects in GO-modified adhesive joints. Wang et al. [70] and Akter et al. [8] confirmed that proper graphene dispersion enhances fracture toughness by promoting crack bifurcation and energy absorption during crack propagation. Moreover, functionalization and controlled exfoliation, as shown by Zabihi et al. [66] and Zotti et al. [72], significantly improve matrix compatibility and reduce stress concentration zones, leading to smoother load transfer and stable crack growth resistance.

In summary, the SEM analysis reveals a progressive transition from brittle to quasi-ductile fracture behavior as the graphene nanoplatelet content and curing temperature are optimized. The 2.0G/9H/120T composite achieves the most favorable balance, exhibiting fine dispersion, controlled crack deflection, and efficient interfacial adhesion, which directly correlates with its outstanding mechanical performance. These observations are consistent with previous findings that identify graphene-induced crack path tortuosity and interfacial debonding as the dominant mechanisms behind the improved fracture resistance of epoxy nanocomposites [71,72,73].

### 3.5. Chemical Analysis

**Figure 15:**
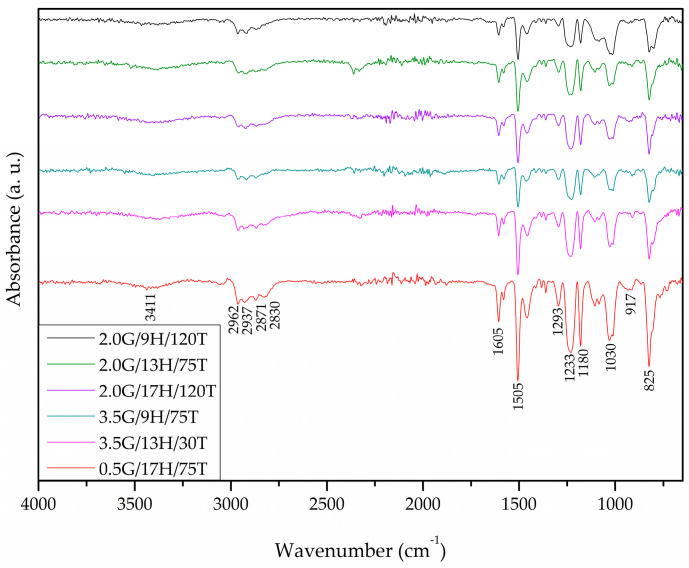
FTIR spectra of composites under different G, H, and T conditions.

FTIR was used to monitor the chemical transformations occurring during the curing of the DGEBA/TETA epoxy system, as well as to assess the influence of GNP and the curing conditions, T and H, on the final chemical structure of the composites. Figure 15 shows the comparative spectra of the samples.

In general, the spectra display the characteristic bands of the aromatic epoxy system, as presented in Table 7, with changes in intensity associated with epoxy ring opening and formation of new ether groups throughout the curing process.

The bands located at 1505 and 1605 cm^−1^ correspond to skeletal vibrations of the aromatic ring of DGEBA [8]. As expected, no significant variations in their intensity were observed, given that the aromatic ring does not participate directly in the curing reactions [74]. However, a slight increase in intensity was found for sample 0.5G/17H/75T, which may be related to physical interaction between well-dispersed GNP sheets and the polymer matrix. This interaction promotes a higher degree of local molecular ordering, resulting in a relative increase in the intensity of this band [75].

The band at 1233 cm^−1^ is associated with the asymmetric axial deformation of the ether group linked to the epoxy ring [8]. A progressive reduction in its intensity was observed as temperature and hardener proportion increased, indicating consumption of the epoxy ring and consequent advancement of the curing reaction [8,74]. This behavior is consistent with the epoxy ring-opening process mediated by the hardener [8].

The band at 1180 cm^−1^ showed behavior similar to that observed at 1233 cm^−1^, with decreasing intensity as the temperature and hardener amount increased. This result reinforces the interpretation that epoxy ring rupture and formation of new linkages occur during curing [8,76].

The characteristic epoxy-ring band, observed at 1295 cm^−1^ [8,77,78], exhibited an abrupt reduction in intensity with increasing temperature and hardener proportion, confirming consumption of the epoxy group throughout the reaction. The decrease of this band constitutes a direct indication of conversion of epoxy groups into hydroxyl and ether groups [76,77].

The band at 1030 cm^−1^, assigned to C–O stretching of aromatic ethers and of ethers formed during curing, showed an increase in intensity with increasing temperature and hardener amount [77,78]. As reported by Vryonis et al. [78], this growth is associated with the formation of new ether groups resulting from epoxy ring opening and secondary reactions between hydroxyl groups and residual epoxy groups.

The deformation band at 917 cm^−1^, attributed to the epoxy (oxirane) ring vibration, shows a marked decrease in intensity and practically disappears after the curing process. This behavior is consistent with the progressive opening of the epoxy ring and its subsequent reaction with the hardener, leading to the formation of hydroxyl and ether groups [74,77,78]. The disappearance of this characteristic band is widely recognized as a reliable indicator of the extent of epoxy conversion and the completion of the crosslinking reaction in DGEBA-based epoxy systems [76].

In addition to the changes associated with the progression of curing, the spectra also reveal specific effects of the presence of GNPs on selected bands. Formulations containing nanoplatelets exhibit a slight increase in the intensity of the aromatic bands at 1505 and 1605 cm^−1^. Although these vibrations do not participate in the chemical curing mechanism, this behavior suggests local modifications in the chemical environment of the DGEBA aromatic rings, possibly related to segmental mobility restrictions induced by well-dispersed graphene nanoplatelets [75,78]. Consistently, some formulations containing 2.0 wt.% GNP also display subtle variations in the relative intensities of the ether band at 1030 cm^−1^, indicating that the nanofiller slightly influences molecular organization in regions close to the interface [78]. No band shifts or the emergence of new functional groups were observed, which is consistent with the chemically inert nature of GNPs and confirms the absence of covalent interactions between the filler and the epoxy matrix [75]. Overall, the spectra reveal subtle yet consistent modifications compatible with non-covalent physical interactions and local mobility restrictions, without indicating any changes in the chemical curing mechanism of the epoxy system [76,77].

The results obtained indicate that higher temperature and increased hardener proportion promote greater advancement of the curing reaction, evidenced by the reduction of bands associated with the epoxy ring (1295 and 917 cm^−1^) and the concomitant increase in the ether band at 1030 cm^−1^. The presence of GNP, in turn, subtly influenced the relative intensities of the aromatic bands (1505–1605 cm^−1^), suggesting physical-chemical interactions between the matrix and conductive nanoparticles, especially in sample 2.0G/9H/120T.

## 4. Conclusions

This study provides a comprehensive understanding of how the synergistic modulation of graphene nanoplatelet content (G), hardener concentration (H), and post-curing temperature (T) governs the multiscale performance of epoxy nanocomposites. Through a statistically structured Box–Behnken Design, nonlinear and interactive effects among these parameters were elucidated, establishing clear correlations between formulation, microstructure, and multifunctional behavior.

Mechanically, the combined optimization of G = 2.0 wt.%, H = 9 phr, and T = 120 °C resulted in exceptional reinforcement efficiency, yielding a flexural strength of 322.0 ± 12.8 MPa, a modulus of 9.7 ± 0.5 GPa, and a strain at break of 4.4 ± 0.2%, representing increases of +197%, +155%, and +91%, respectively, compared with neat epoxy. These results surpass those of conventional GO- and CNT-filled systems, highlighting that the mechanical improvement arises not only from the inherent stiffness of GNPs but also from the synergistic balance between crosslink density (controlled by H and T) and nanoparticle dispersion (governed by G).

The dynamic mechanical response confirmed this synergy as the storage modulus rose from 2.9 GPa to 7.5 GPa and the glass transition temperature (Tg) reached 143 °C, evidencing restricted molecular mobility and enhanced network stiffness. The post-curing temperature (T) emerged as the dominant factor controlling viscoelastic reinforcement, while the optimized GNP concentration (G) improved interfacial load transfer without inducing aggregation. The balance of hardener content (H) was critical to sustain toughness, preventing embrittlement at higher crosslink densities.

The thermal analyses (TGA and DSC) revealed a 15 °C shift in degradation onset, confirming that the GNPs act as thermal barriers by delaying heat and mass transport during degradation. These gains were maximized under higher post-curing temperatures (T = 120 °C), which promoted network densification and reduced unreacted epoxy groups, as verified by FTIR through the attenuation of the epoxide (≈915 cm^−1^) and hydroxyl absorption bands. The FTIR results thus corroborate the completion of the curing reaction and the enhanced crosslinking efficiency promoted by GNP addition.

From a morphological perspective, SEM and TEM analyses showed uniform dispersion and strong interfacial adhesion between GNPs and the epoxy matrix, while AFM confirmed the presence of few-layer nanoplatelets (2–8 nm) with well-defined lateral dimensions. This microstructural arrangement underlies the observed improvements in stiffness, thermal stability, and viscoelastic damping.

It should be noted that the present BBD optimization was carried out using a single commercial GNP grade, characterized by few-layer nanoplatelets (2–8 nm thick) with well-defined lateral dimensions. Therefore, the quantitative values of the optimized properties are specific to this morphology. Nevertheless, the qualitative trends identified here are expected to hold for other 2D carbon nanofillers; higher-aspect-ratio and thinner nanoplatelets would, in principle, provide more efficient load transfer and may require lower contents to achieve similar stiffness and damping enhancements, whereas more oxidized or functionalized platelets (approaching GO-like chemistry) could increase interfacial bonding and constrained interphase volume at the expense of some intrinsic stiffness and thermal stability. Conversely, thicker, less exfoliated GNP grades would likely require higher loadings and might reduce the reinforcement efficiency due to aggregation and lower effective aspect ratio.

Overall, the integration of statistical design, spectroscopic validation, and multiscale characterization provides a predictive framework that transcends empirical optimization. The findings demonstrate that performance enhancement in GNP/epoxy systems is dictated more by parameter synergy than by filler content, representing a paradigm shift for the design of lightweight, high-strength, and thermally robust polymers. The proposed approach is versatile and scalable, enabling tailored formulations for aerospace, protective coatings, and energy storage applications. However, some practical limitations should be acknowledged. Even at the relatively low optimized GNP content, adequate dispersion and degassing are required to avoid agglomerates and voids, which may demand high-shear mixing or vacuum-assisted processing at larger scales. In addition, the highly crosslinked network that maximizes stiffness and Tg can increase brittleness, potentially limiting applications that require high impact resistance or pronounced ductility, and the use of nanofillers also introduces additional cost and quality-control requirements associated with filler handling and consistency. These aspects, together with long-term durability and fatigue performance, should be addressed in future studies aimed at specific end-use implementations.

## Figures and Tables

**Table 1:** Factors and corresponding levels investigated in this study.

c_i_	GNP Content (G) (wt.%)	Hardener Content (H) (wt.%)	Post-Curing Temperature (T) (°C)
−1	0.5	9	30
0	2.0	13	70
+1	3.5	17	120

**Table 2:** BBD model parameters and sample nomenclature.

	Coded Parameters			Uncoded Parameters			
Number	G	H	T	G	H	T	Nomenclature
1	−1	−1	0	0.5	9	75	0.5G/9H/75T
2	1	−1	0	3.5	9	75	3.5G/9H/75T
3	−1	1	0	0.5	17	75	0.5G/17H/75T
4	1	1	0	3.5	17	75	3.5G/17H/75T
5	−1	0	−1	0.5	13	30	0.5G/13H/30T
6	1	0	−1	3.5	13	30	3.5G/13H/30T
7	−1	0	1	0.5	13	120	0.5G/13H/120T
8	1	0	1	3.5	13	120	3.5G/13H/120T
9	0	−1	−1	2.0	9	30	2.0G/9H/30T
10	0	1	−1	2.0	17	30	2.0G/17H/30T
11	0	−1	1	2.0	9	120	2.0G/9H/120T
12	0	1	1	2.0	17	120	2.0G/17H/120T
13	0	0	0	2.0	13	75	2.0G/13H/75T

**Table 3:** Summary of the thermal properties of the nanocomposites.

Uncoded Parameters			Thermal Properties			
G	H	T	Tg (°C)	Tonset (°C)	Tmax (°C)	ΔH_res_ (J.s^−1^)
0.5	9	75	83.3	350.2	385.1	0.4
3.5	9	75	80.4	350.1	380.7	0.9
0.5	17	75	93.7	344.2	370.4	2.4
3.5	17	75	89.4	342.2	373.3	1.7
0.5	13	30	54.9	343.4	369.3	6.5
3.5	13	30	54.8	342.2	371.1	9.2
0.5	13	120	114.5	345.2	382.0	-
3.5	13	120	106.3	352.4	374.4	-
2.0	9	30	49.4	342.2	372.7	5.1
2.0	17	30	54.0	344.6	370.8	8.9
2.0	9	120	118.0	347.4	375.3	-
2.0	17	120	109.7	343.0	371.8	-
2.0	13	75	87.9	346.0	376.6	0.9

- Not observed.

**Table 4:** Summary of the flexural properties of the nanocomposites.

Uncoded Parameters			Mechanical Properties		
G	H	T	MOR (MPa)	MOE (GPa)	ε_f_ (%)
0.5	9	75	205.2 ± 8.5	10.1 ± 0.3	2.6 ± 0.1
3.5	9	75	284.8 ± 6.4	12.1 ± 0.1	2.8 ± 0.1
0.5	17	75	285.0 ± 10.6	9.2 ± 0.3	3.7 ± 0.2
3.5	17	75	229.4 ± 19.7	8.7 ± 0.1	3.0 ± 0.2
0.5	13	30	216.3 ± 5.8	11.8 ± 0.6	2.2 ± 0.1
3.5	13	30	213.0 ± 9.2	11.8 ± 0.2	2.1 ± 0.1
0.5	13	120	259.2 ± 9.6	8.6 ± 0.1	3.8 ± 0.2
3.5	13	120	245.8 ± 18.9	9.1 ± 0.1	3.5 ± 0.3
2.0	9	30	240.9 ± 32.8	12.6 ± 1.0	2.3 ± 0.2
2.0	17	30	180.7 ± 6.7	10.8 ± 0.2	1.9 ± 0.1
2.0	9	120	322.0 ± 12.8	9.7 ± 0.5	4.4 ± 0.2
2.0	17	120	245.8 ± 5.9	8.3 ± 0.4	3.9 ± 0.1
2.0	13	75	253.6 ± 35.7	10.6 ± 0.7	2.8 ± 0.3

**Table 5:** Flexural properties of the optimized formulation in relation to the composite in the literature.

System	Filler Content	MOR (MPa)	MOE (GPa)	ΔMOR (%)	ΔMOE (%)	References
Neat epoxy (DGEBA/TETA)	0 wt.%	70–110	2.2–3.0	+258	+273	[36,37,38]
Epoxy + palm fibers	50 vol.%	32.64	3.28	+887	+196	[36]
Epoxy + jute fibers	50 wt.%	208–248	2.0–2.5	+41	+331	[39]
Epoxy + bamboo fibers + GO	40–50 vol.%	259.9–334.6	16.7–23.8	+8	−52	[40]
Epoxy + GO	0.5–1.0 wt.%	98.5	3.65	+227	+166	[38]
Epoxy + rGO	0.5–1.0 wt.%	92.7	3.28	+247	+196	[38]
Epoxy + graphite	1.0–1.2 wt.%	80–90	3.0–3.2	+279	+213	[41]
Pristine graphene/epoxy	0.5–1.0 wt.%	118.9	3.41	+171	+185	[42]
GO/aramid fiber/epoxy	0.1–0.7 wt.%	130.5	2.94	+147	+230	[43]
Epoxy + gNP	0.5–1.5 wt.%	80–110	2.8–3.2	+239	+223	[44,45]
Epoxy+ NH_2_-GNs	0.6 wt.%	152.3	5.4	+111	+96	[46]
Epoxy + pDop-rGO	0.1 wt.%	141	5.3	+128	+83	[47]
**Epoxy + gNP (optimized)**	**2.0 wt.% GNP** **(2.0G/9H/120T)**	**322.0**	**9.7**	—	—	**PW**

PW—Present Work. Bold values indicate the optimized composite.

**Table 6:** Viscoelastic parameters obtained from DMA for the GNP-reinforced epoxy nanocomposites, showing the effects of GNP content (G), hardener concentration (H), and post-curing temperature (T) on Tg, E′, tanδ_max_, ΔlogE′, and crosslink density (ν_e_).

Uncoded Parameters			Viscoelastic Properties					
G	H	T	Tg (°C)	E′ (30 °C) (MPa)	E′ (Tg + 30 °C) (MPa)	ΔlogE′	tanδ_max_	$v_{e}$ (×10^−3^ mol.m^−3^)
0.5	9	75	94.1	3141.2	31.4	2.0	0.8	3.2
3.5	9	75	104.7	4046.3	47.2	1.9	0.8	4.6
0.5	17	75	116.4	2616.7	59.6	1.6	0.4	5.7
3.5	17	75	115.3	2835.4	60.0	1.7	0.5	5.7
0.5	13	30	82.2	2473.6	65.9	1.6	1.0	6.9
3.5	13	30	81.8	3659.7	136.8	1.4	0.9	14.2
0.5	13	120	138.2	2140.5	38.8	1.7	0.5	3.5
3.5	13	120	139.5	2521.5	53.7	1.7	0.5	4.9
2.0	9	30	72.9	3144.3	9.4	2.5	1.1	1.0
2.0	17	30	132.0	2770.1	49.1	1.8	0.5	4.5
2.0	9	120	133.0	2904.0	46.0	1.8	0.6	4.2

**Table 7:** Vibrational band assignments obtained from FTIR analysis of the epoxy systems.

Vibration	Wavenumber (cm^−1^)
-OH stretching	3411
C-H stretching	2800–3000
Asymmetrical stretching -CH and -CH_2_ group	2937
Symmetrical stretching -CH and -CH_3_ group	2871
C=C stretching bands of aromatic rings	1505–1605
Epoxy ring mode	1295, 917
Asymmetric axial deformation of the ether group	1233
C-O stretching of the aromatic ring	1180
Symmetrical aromatic C-O stretching	1030
Aromatic ring bent out of plane	825

## Data Availability

The original contributions presented in this study are included in the article. Further inquiries can be directed to the corresponding author.
